# Diagnostic approaches to Kawasaki disease worldwide: the results from the JIR-CliPS network

**DOI:** 10.1093/rheumatology/keag340

**Published:** 2026-06-26

**Authors:** Jurgita Marčiulynaitė, Daiva Gorczyca, Aušra Šnipaitienė, Konstantinos Pateras, Teresa Giani, Margarita Ganeva, Zeynep Balik, Francois Hofer, Marie Frank, Jaanika Ilisson, Jacek Postepski, Judith Sánchez-Manubens, Arūnė Ramanauskienė, Cristina Costa Duarte Lanna, Jacqueline Yan, Dzifa Dey, Seza Ozen, Michael Hofer, Yosef Uziel, Tilmann Kallinich

**Affiliations:** Department of Paediatrics, Lithuanian University of Health Sciences, Kaunas, Lithuania; Department of Paediatric Respiratory Medicine, Immunology and Critical Care Medicine, Charité—Universitätsmedizin Berlin, Corporate Member of Freie Universität Berlin and Humboldt-Universität, Berlin, Germany; Department of Paediatrics, Lithuanian University of Health Sciences, Kaunas, Lithuania; Department of Statistics, Athens University of Economics and Business, Athens, Greece; Department of Paediatrics, AOU Meyer IRCCS, Florence, Italy; Department of Paediatric Rheumatology, Medical University Sofia, Sofia, Bulgaria; Department of Paediatric Rheumatology, Trabzon Kanuni Training and Research Hospital, Trabzon, Turkey; Fondation Rhumatismes-Enfants-Suisse, Lausanne, Switzerland; Fondation Rhumatismes-Enfants-Suisse, Lausanne, Switzerland; Department of General Paediatrics and Neurology, Tartu University Hospital, Children’s Clinic, Tartu, Estonia; Department of Paediatric Pulmonology and Rheumatology, Medical University of Lublin, Lublin, Poland; Paediatric Rheumatology Unit, Parc Taulí Hospital Universitari. Institut d’Investigació i Innovació Parc Taulí (I3PT-CERCA), Sabadell, Spain; Gijos Clinics, Kaunas, Lithuania; Locomotor System Department, Rheumatology Unit, Universidade Federal of Minas Gerais, Belo Horizonte, Minas Gerais, Brazil; Department of Paediatric Rheumatology, Starship Children’s Health and University of Auckland, Auckland, New Zealand; Rheumatology Unit, Department of Medicine and Therapeutics, University of Ghana Medical School, Accra, Ghana; Department of Paediatric Rheumatology, Hacettepe University, Ankara, Turkey; Fondation Rhumatismes-Enfants-Suisse, Lausanne, Switzerland; Department of Paediatrics, Hôpital Riviera-Chablais, Rennaz, Switzerland; Department of Paediatrics, Meir Medical Centre, Kfar Saba, Gray School of Medicine, Tel Aviv University, Tel Aviv, Israel; Department of Paediatric Respiratory Medicine, Immunology and Critical Care Medicine, Charité—Universitätsmedizin Berlin, Corporate Member of Freie Universität Berlin and Humboldt-Universität, Berlin, Germany; German Rheumatism Research Centre, An Institute of the Leibniz Association, Berlin, Germany; German Centre for Child and Adolescent Health (DZKJ), Partner Site Berlin, Berlin, Germany

**Keywords:** Kawasaki disease, diagnostic approaches, coronary artery, worldwide, clinical practice, survey

## Abstract

**Objectives:**

To evaluate worldwide differences of diagnostic approaches to Kawasaki disease (KD), focusing on aspects, which vary throughout major international guidelines, including criteria for diagnostic and risk stratification.

**Methods:**

An online English-language survey was distributed to physicians worldwide between June 2022 and November 2024 through the JIR-CliPS (juvenile inflammatory rheumatism; clinical practice strategies) network, aiming to collect real-life diagnostic and management practices in juvenile rheumatic conditions, including KD. Responses addressing the definitions of complete and incomplete KD and criteria identifying patients at high-risk for coronary artery lesions (CALs), were analysed. Descriptive statistics, comparisons of categorical variables, sensitivity and weighted analysis were performed.

**Results:**

One hundred and ninety-two physicians from 51 countries completed the survey. All respondents accepted ≥5 days of fever plus four criteria as diagnostic for complete KD. Thirty-two percent (*n* = 62/192, 95% CI [25.74, 39.40]) also accepted shorter fever duration, particularly physicians from France (*n* = 16/24, 63%, 95% CI [44.68, 84.37]). Ninety-four percent (*n* = 178/189, 95% CI [89.83, 97.06]) diagnosed incomplete KD based on fever and three clinical criteria. Forty-four percent (*n* = 84/189, 95% CI [37.23, 51.83]) accepted the presence of CALs as diagnostic in febrile patients, even without other clinical criteria; this approach was more common among Europeans. Infant age and elevated coronary *Z*-scores were identified as key indicators for coronary involvement. Years of experience did not influence the results.

**Conclusion:**

Despite shared core diagnostic principles, country-specific differences persist in real-life clinical practice regarding fever duration, minimum clinical criteria and the role of coronary artery findings in diagnosing complete and incomplete KD. Future real-life studies are needed to support timely and consistent diagnosis.

## Graphical abstract

**Graphical Abstract:**
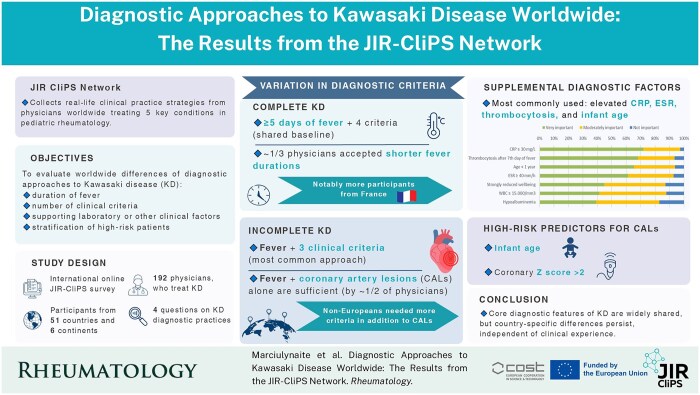

Rheumatology key messages

- About ≥5 febrile days with four criteria prove complete Kawasaki Disease (KD); one-third accept shorter fevers, particularly those who are French.
- Incomplete KD is commonly diagnosed with three criteria; half consider fever with coronary lesions sufficient.
- Infancy and coronary Z-score >2 are major risk factors for coronary aneurysms in KD worldwide.

## Introduction

Kawasaki disease (KD) is an acute self-limiting systemic vasculitis, commonly affecting small- and medium-sized vessels in early childhood [1] and is the leading cause of childhood-acquired heart disease in developed countries [2]. Necrotizing vasculitis may result in severe coronary artery lesions (CALs), occurring in up to 20–25% of untreated patients [3]. However, timely applied IVIG can reduce the incidence of CALs to ∼4% [3]. Extra-coronary arteries, such as brachial or iliac arteries, may also be involved, primarily in younger infants [4]. Mortality rate is low (<0.25%), with the peak fatality occurring within the first 2 months after the onset of fever, and almost exclusively resulting from cardiovascular events [5].

KD diagnosis still relies on the clinical presentation supported by complementary laboratory and imaging tests. Unfortunately, a specific biomarker for KD currently does not exist [6]. Although recommendations for KD from several international and national paediatric societies are updated regularly, discrepancies, including differences in establishing the diagnosis, persists [3, 6–8]. Although previous studies revealed international real-life differences in the therapeutic management of KD [9–11], comprehensive analysis of country-specific diagnostical strategies are lacking.

Given the importance of accurate diagnosis and prompt treatment together with the vigilance for over-diagnostics and over-treatment, identifying high-risk patients for coronary aneurysms or resistance to primary treatment should be prioritized. However, the criteria to identify patients with a high-risk profile vary across guidelines and often depend on ethnicity-specific scoring systems [6, 12–14]. The risk of CALs decreases with timely and potent adjunctive corticosteroid therapy [15, 16] which is increasingly used in cases of incomplete KD or in high-risk groups. But the optimal steroid regimens remain uncertain [17]. In high-risk patients, alternative first-line or adjunctive therapies, including ciclosporin or infliximab, can prevent or resolve CALs [18, 19], while trials with interleukin-1 blockers are ongoing [20, 21].

The medical guidelines are based on high-quality scientific data and/or expert consensus but their interpretation and implementation may vary between clinicians and across countries. For example, a needs assessment survey of rheumatologists caring for children with ANCA-associated vasculitis showed that only 25% of respondents used EULAR/EUVAS recommendations to guide their treatment decisions [22]. Similarly, substantial variability in the management of acute KD has also been reported [11].

The main objective of JIR (juvenile inflammatory rheumatism) network is to collect real-life clinical practice strategies (CliPS) from physicians all over the world taking care of patients with juvenile inflammatory conditions into a library and compare the management strategies to future implementation in differential healthcare systems.

The aim of this study was to investigate how physicians worldwide define and diagnose complete and incomplete KD, as well as identify high-risk patients, and to compare these diagnostic practices across countries and continents using data from the international JIR-CliPS survey.

## Methods

### Design and population

The JIR-CliPS project focuses on five rheumatic conditions, including: (i) LN; (ii) IgA vasculitis (IgAV) and KD; (iii) biologics in autoinflammatory diseases; (iv) periodic fever, aphthous stomatitis, pharyngitis and adenitis (PFAPA) syndrome, and syndrome of undifferentiated recurrent fever (SURF) and (v) Still’s disease.

The questionnaire, which included questions on demographics, disease-specific diagnostical and treatment approaches and country-specific resources, was developed by the core project group between October 2021 and May 2022, and was then validated in a pilot study conducted from June to September 2022 across four paediatric rheumatology centres: Accra (Ghana), Auckland (New Zealand), Belo Horizonte (Brazil) and Lausanne (Switzerland). Based on feedback from the pilot study, the final questionnaire was launched online in January 2023 using the Eval&GO^®^ platform. Data were collected from the pilot phase until November 2024. The survey targeted physicians dealing with vasculitis. Participants were recruited internationally via email and professional networks, including conferences and learning societies. They completed the survey using a secure personal link.

Participants provided informed consent by voluntarily completing and submitting the questionnaire. Personal identifiers were requested for clarification but were inaccessible during analysis, following a trustee principle. The study adhered to the Declaration of Helsinki and the General Data Protection Regulation (GDPR). Ethics approval was deemed unnecessary due to the non-invasive design and the absence of sensitive data.

### Measures

The Vasculitis working group survey comprised 20 questions for KD, and 20 questions for IgAV. The survey included close-ended questions, providing single or multiple-choice answers. Questions about KD were divided into three sections: (1) diagnosis, (2) initial therapy and (3) refractory cases, including IVIG resistance and prolonged/late presentation of KD.

This study analysed four questions addressing diagnostic patterns in KD and the identification of patients at high-risk of CALs (Supplementary Data S1). Two questions asked participants to rate factors supporting the diagnosis of incomplete KD and the assignment of patients to high-risk of CALs using an ordinal scale ranging from ‘not important’ to ‘very important’. To summarize physicians’ ratings, factors were grouped into structure-based importance categories. For factors supporting the diagnosis of incomplete KD, three importance levels were defined: (I) >50% of respondents rated the factor as ‘very important’; (II) more respondents rated a factor as ‘very important’ and ‘moderately important’ rather than ‘moderately’ and ‘not important’; (III) all remaining factors (when less respondents rated the factor as ‘very important’ and ‘moderately important’ rather than ‘moderately’ and ‘not important’). For factors identifying patients at high-risk of CALs, four importance levels were defined: (I) >50% of respondents rated the factor as ‘very important’; (II) the combined proportion of ‘very important’ and ‘important’ ratings exceeded that of all lower ratings (‘moderately’, ‘slightly’ and ‘not important’); (III) the combined proportion of ‘very important’, ‘important’ and ‘moderately important’ ratings exceeded that of lower ratings and (IV) all remaining factors, which were predominantly rated as ‘slightly’ or ‘not important’.

### Statistical analysis

Pseudonymized results were exported to Microsoft Excel. Analysis was performed using Microsoft Excel (Version 2019, Microsoft, Redmond, Washington) and R (Version 4.5.1, R Foundation for Statistical Computing, Vienna, Austria). For the primary comparative analyses (Europe *n* = 109 vs non-Europe *n* = 83), the study had 80% power to detect a 15% difference in proportions at a 5% significance level. Categorical variables were compared using the *χ*^2^ test or Fisher’s exact test. Proportions were reported with 95% CIs. Available-case basis was used for each question; denominators varied across questions. Sensitivity and weighted analyses were undertaken. Statistical significance was defined as a *P-*value <0.05.

## Results

Responses from 192 physicians representing 51 countries and six continents were collected, mostly from Europe (*n* = 109, 57%). Most physicians were from France (*n* = 24, 13%), Turkey (*n* = 21, 11%), Brazil (*n* = 14, 7%), Spain (*n* = 13, 7%) and Germany (*n* = 10, 5%). All participating countries are illustrated in Supplementary Fig. S1. Paediatric rheumatologists constituted the majority of respondents (*n* = 138, 72%). Almost all participants (*n* = 180, 94%) were involved in inpatient and outpatient care and most (*n* = 169, 88%) treated primarily children. Demographic characteristics are shown in Table 1.

**Table 1:** Demographic characteristics of respondents (*n* = 192).

	Number of respondents (%)
Gender	
Female	140 (72.9)
Male	51 (26.6)
Did not wish to answer	1 (0.5)
Kind of institution	
University hospital	131 (68.2)
Tertiary hospital	24 (12.5)
Hospital	29 (15.1)
Private practice	8 (4.2)
Kind of practice	
Inpatient	3 (1.6)
Outpatient	9 (4.7)
Both	180 (93.8)
Kind of patients	
Only children	169 (88.0)
Only adults	6 (3.1)
Both children and adults	16 (8.3)
Speciality	
Paediatric rheumatology	138 (71.9)
Paediatrician	64 (33.3)
Paediatric immunology	24 (12.5)
Adult rheumatology	13 (6.8)
Paediatric cardiology	10 (5.2)
Paediatric infectious disease specialist	9 (4.7)
Paediatric nephrology	7 (3.6)
Primary care paediatrician	4 (2.1)
Adult immunology	3 (1.6)
Dermatologist	1 (0.5)
Adult nephrology	1 (0.5)
Experience in paediatric vasculitis	
0–4 years	32 (16.7)
5–9 years	57 (29.7)
>10 years	103 (53.6)
Respondents’ distribution by continent	
Europe (27 countries)	109 (56.8)
Asia (eight countries)	36 (18.8)
North and South America (eight countries)	25 (13.0)
Africa (seven countries)	17 (8.9)
Australia and Oceania (one country)	5 (2.6)

### Diagnosis of complete KD

In our survey, all respondents (*n* = 192) considered ≥5 days of fever with four clinical criteria sufficient for the diagnosis of complete KD. However, 32% (*n* = 62/192, 95% CI [25.74, 39.40]) accepted a diagnosis at 4 days of fever and 16% (*n* = 31/192, 95% CI [11.24, 22.13]) even at 3 days.

**Figure 1:**
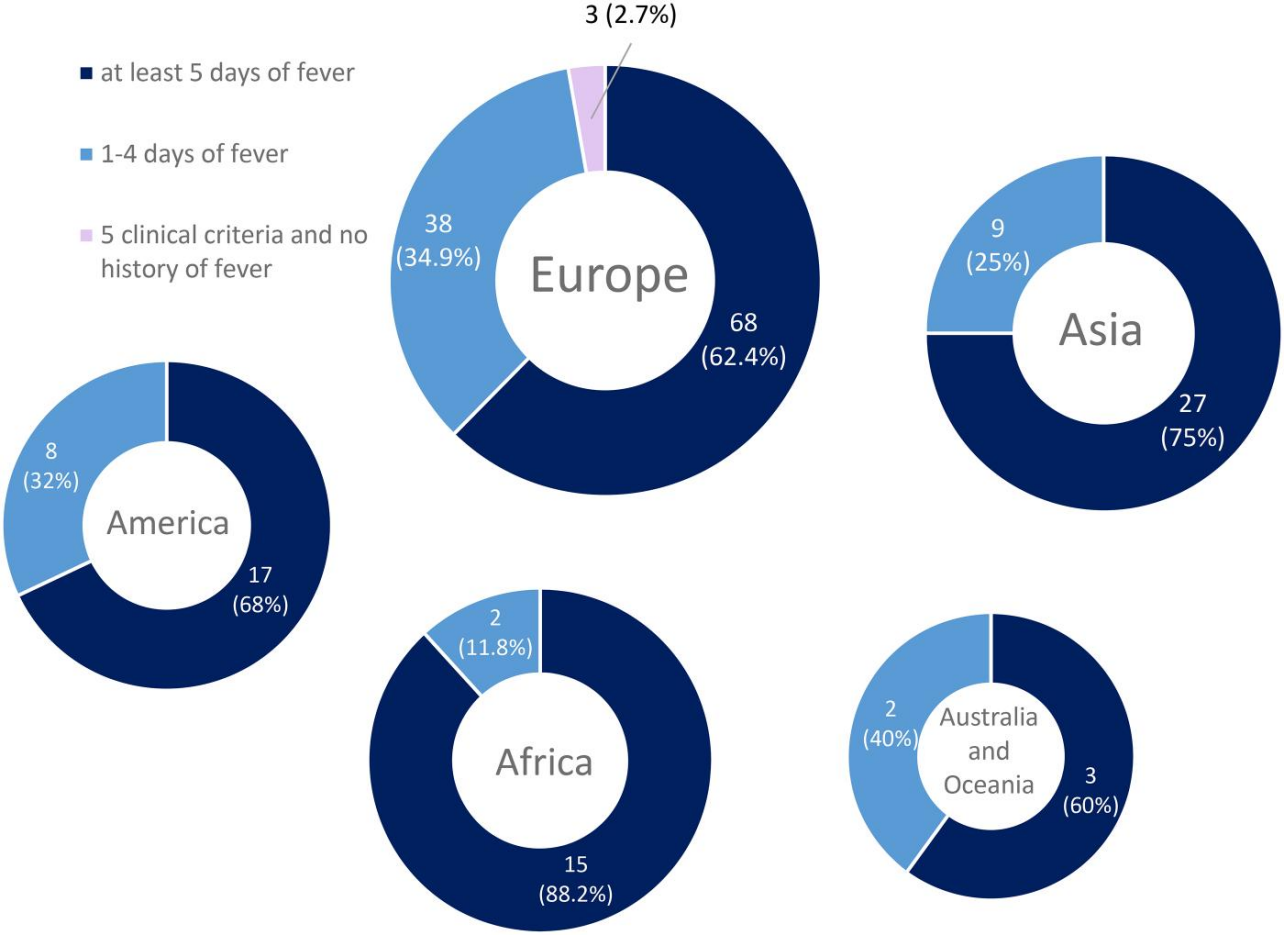
Duration of fever required for the diagnosis of complete Kawasaki disease across continents (*n* = 192)

Acceptance of shorter fever durations (<5 days) varied by region and was more frequent in Europe (*n* = 41/109, 38%, 95% CI [28.52, 47.40]) than in Asia (*n* = 9/36, 25%, 95% CI [12.12, 42.20]) and Africa (*n* = 2/17, 12%, 95% CI [1.46, 36.44]). Although the difference between European and non-European physicians was not statistically significant (38% (*n* = 41/109) vs 25% (*n* = 21/83), *χ*^2^  *P* = 0.071), a 13% absolute difference suggests potential variation (95% CI [−0.7, 25.3]). The distribution of responses by continent is shown in Fig. 1.

**Figure 2:**
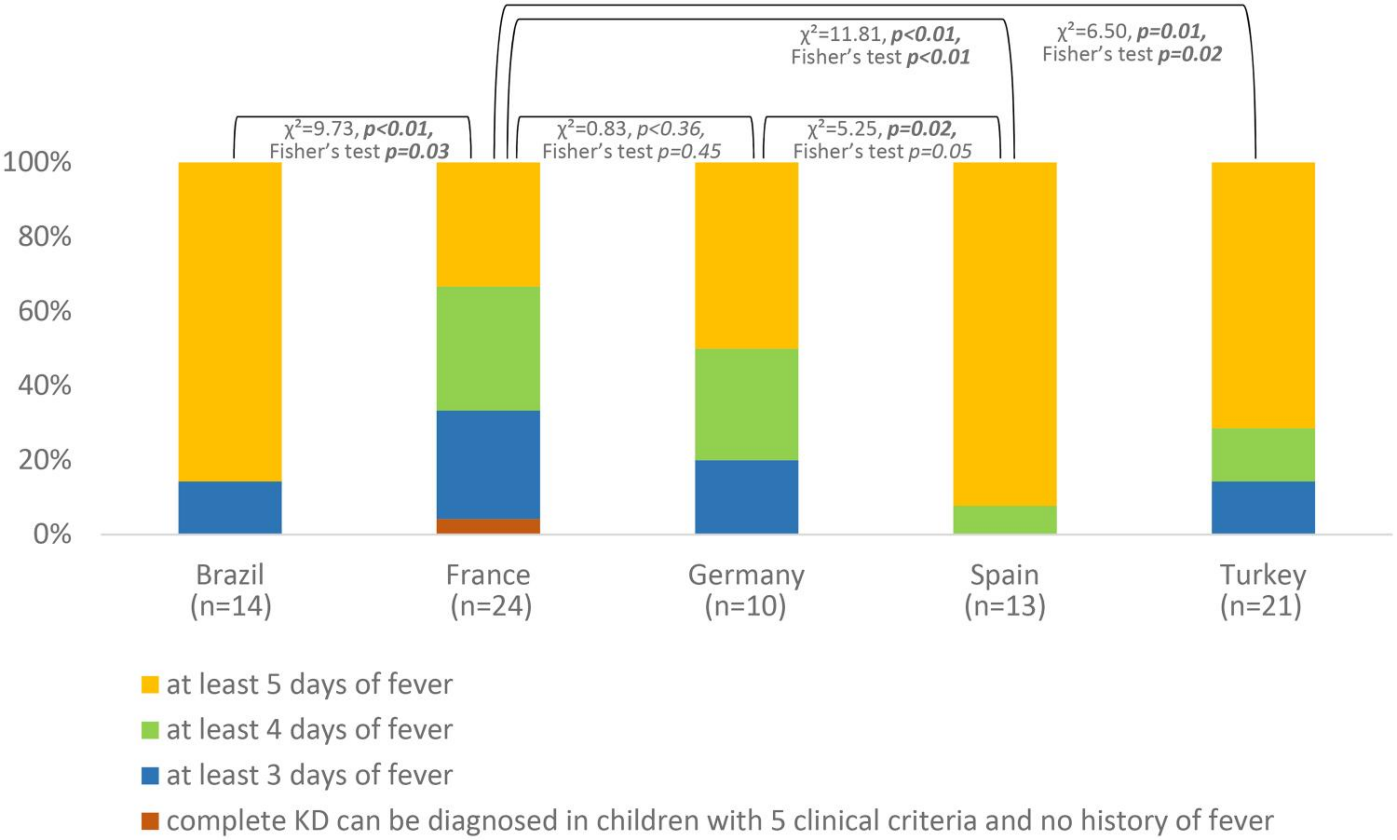
Necessary number of febrile days to diagnose complete Kawasaki disease according to physicians from most represented countries (statistically significant differences between countries are highlighted in bold; note that statistical significance persists using Fisher’s exact test, except a difference between Germany and Spain is not statistically significant using Fisher’s exact test). KD, Kawasaki disease

A comparison of the five most represented countries (Brazil, France, Germany, Spain and Turkey) revealed national differences in the acceptance of shorter fever duration for the diagnosis of complete KD. An overall country comparison showed a significant difference in country-level diagnostic patterns (*χ*^2^  *P* = 0.001). French physicians more often accepted ≤4 days of fever (*n* = 16/24, 63%, 95% CI [44.68, 84.37]), whereas German physicians were evenly divided (*n* = 5/10, 50%, 95% CI [18.7, 81.3]). In contrast, physicians from Turkey, Spain and Brazil mostly required ≥5-day fever (Fig. 2).

Stratification by years of clinical experience showed no clear patterns in the diagnosis of complete KD before five febrile days between physicians with ≤4, 5–9, and ≥10 years’ experience (36% (*n* = 37/103, 95% CI [26.70, 45.97]) vs 25% (*n* = 8/32, 95% CI [11.46, 43.40]) and 30% (*n* = 17/57, 95% CI [18.43, 43.40]); *χ*^2^  *P* = 0.459).

### Diagnosis of incomplete KD

When initial work-up does not suggest an alternative diagnosis and CALs are absent, 94% (*n* = 178, 95% CI [89.83, 97.06]) of physicians diagnosed incomplete KD in patients with fever ≥5 days and three clinical criteria. Fewer respondents diagnosed incomplete KD with one or two criteria (11% (*n* = 21, 95% CI [7.01, 16.48]) and 50% (*n* = 94, 95% CI [42.40, 57.08]), respectively). Physicians from Turkey and Germany were more likely to diagnose incomplete KD with one or two clinical criteria: 24% (*n* = 5/21, 95% CI [8.22, 47.17]) and 20% (*n* = 2/10, 95% CI [2.52, 55.61]) diagnosed KD with one, and 64% (*n* = 13/21, 95% CI [38.44, 81.89]) and 60% (*n* = 6/10, 95% CI [26.24, 87.84]) with two, respectively. No statistically significant differences were observed when comparing diagnostic acceptance for one or two criteria plus fever vs three criteria plus fever, either among the five most represented countries (*χ*^2^  *P* = 0.859) or across continents (*χ*^2^  *P* = 0.682).

All respondents (*n* = 189) considered a combination of fever and three clinical criteria, as well as CALs, to be sufficient for the diagnosis of incomplete KD. Furthermore, 44% (*n* = 84, 95% CI [37.23, 51.83]) of physicians would diagnose incomplete KD based on fever and CALs alone, even in the absence of other clinical criteria. The majority of respondents who accepted this approach were from Europe (*n* = 52/106, 49%, 95% CI [39.22, 58.95]), with the highest proportion of these being from Germany (*n* = 7/10, 70%, 95% CI [34.75, 93.33]), and the lowest from Africa (*n* = 5/17, 29%, 95% CI [10.31, 55.96]). These differences were not statistically significant among the most represented countries (*χ*^2^  *P* = 0.298) or across continents (*χ*^2^  *P* = 0.361). In the presence of CALs, the diagnostic thresholds differed between European and non-European respondents (*χ*^2^  *P* = 0.047), with non-European respondents requiring more additional criteria to diagnose incomplete KD.

In the absence of a fever, only 16% (*n* = 31/189, 95% CI [11.42, 22.47]) of respondents would diagnose KD in patients who fulfil four out of five clinical criteria. Very few physicians diagnose KD when only three or two criteria are present and no fever occurs (5% (*n* = 9/189, 95% CI [2.20, 8.85]) and 2% (*n* = 3/189, 95% CI [0.33, 4.57]), respectively). Acceptance of a diagnosis based on clinical criteria (ranging from two to four) in the absence of fever was most prevalent in Europe (*n* = 22/106, 21%, 95% CI [13.49, 29.72]), particularly in Germany (*n* = 5/10, 50%, 95% CI [18.71, 81.29]), compared with Africa (*n* = 2/17, 12%, 95% CI [1.46, 36.44]), Asia (*n* = 3/36, 8%, 95% CI [1.75, 22.47]) and the Americas (*n* = 1/25, 4%, 95% CI [0.10, 20.35]). The observed variations were independent of physicians’ experience (Supplementary Fig. S2).

### Additional factors supporting the diagnosis of incomplete KD

**Figure 3:**
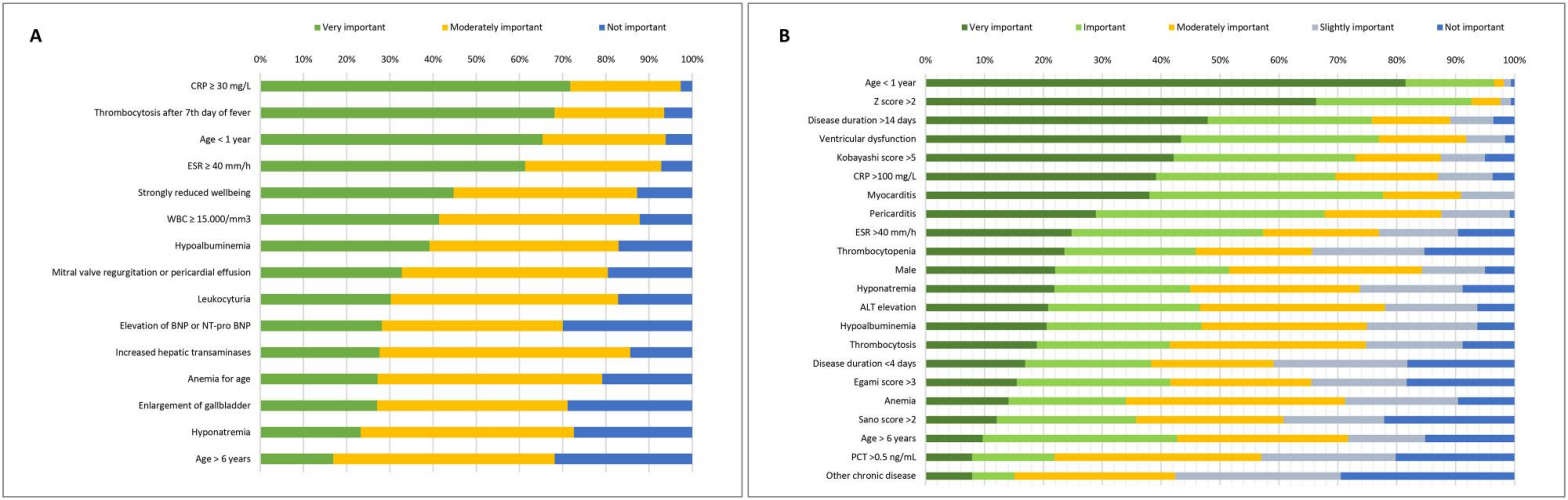
Clinical and laboratory factors for diagnosis and risk stratification in Kawasaki disease. (**A**) Factors supporting the diagnosis of incomplete Kawasaki disease (*n* = 190). (**B**) Factors associated with a high-risk of coronary artery lesions (*n* = 176). ALT, alanine aminotransferase; BNP, brain natriuretic peptide; NT-pro BNP, N-terminal pro-brain natriuretic peptide; PCT, procalcitonin; WBC, white blood cell count

Physicians most frequently reported the following factors as ‘very important’: CRP >30 mg/l, thrombocytosis after day 7, age <1 year and ESR >40 mm/h (Fig. 3A). Those more commonly rated as ‘moderately important’ included anaemia, hypoalbuminaemia, white blood cell (WBC) count of ≥15 000/mm³, leukocyturia, increased hepatic transaminases, mitral valve regurgitation or pericardial effusion and strongly reduced well-being.

### Features identifying patients at high-risk for CALs

Based on respondents rating, these features were categorized into four levels of importance. Age <1 year (*n* = 141/173, 82%) and *Z*-score >2 (*n* = 108/163, 66%) were assigned to the highest importance level (Fig. 3B). Features classified as second-level importance included CRP >100 mg/l, ESR >40 mm/h, Kobayashi score >5, pericarditis, myocarditis, ventricular dysfunction, disease duration >14 days and male gender. Anaemia, hypoalbuminemia, hyponatremia, thrombocytosis, thrombocytopenia, elevated alanine aminotransferase (ALT), Egami score >3 and age >6 years were assigned third-level importance. Several factors, including the presence of other chronic diseases, Sano score >2, procalcitonin (PCT) >0.5 ng/ml and disease duration less than 4 days, were consistently rated as having low importance.

**Figure 4:**
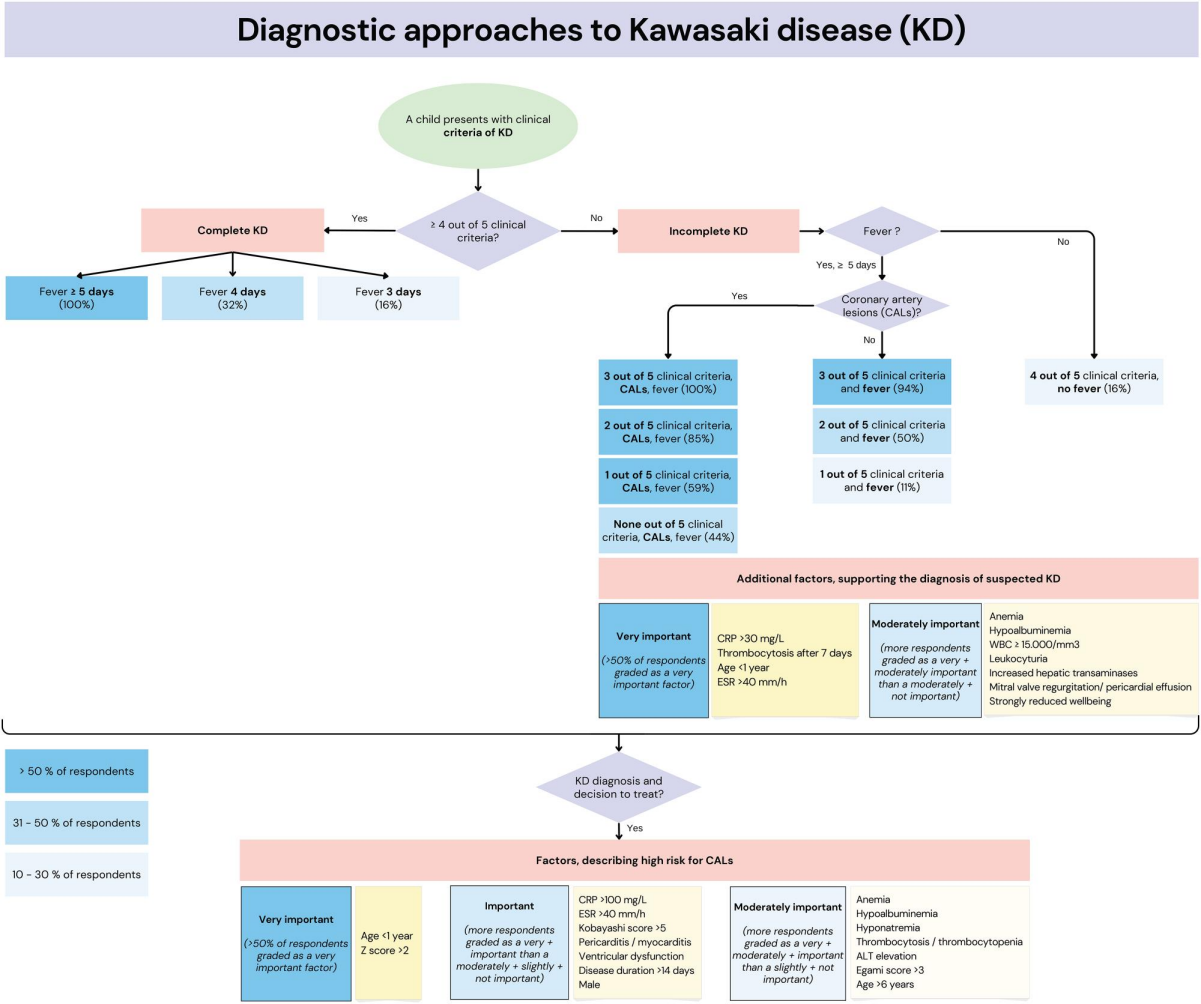
Diagnostic approaches to Kawasaki disease according to JIR-CliPS survey. CALs, coronary artery lesions; KD, Kawasaki disease; WBC, white blood cell count

Diagnostic strategies reported by the physicians in this survey are summarized in Fig. 4, with respondent distributions further stratified by continent, country and years of clinical experience in Supplementary Fig. S2.

### Comments on data reliability

To strengthen the reliability of the data, we performed a sensitivity analysis by sequentially excluding each of the five countries with the largest representation and recalculating the response frequencies for each question. No meaningful changes to the overall response pattern were observed when any or all of the five overrepresented countries (France, Turkey, Brazil, Germany and Spain) were excluded. The detailed results of the sensitivity analysis are shown in Supplementary Data S2.

Additionally, we performed a weighted analysis, assigning a weight (coefficient) to each represented country based on physician density (data available from https://www.who.int/data/gho/data/indicators/indicator-details/GHO/medical-doctors-(per-10-000-population)). The results of the weighted analysis are shown in Supplementary Data S3. These results suggest that our findings are robust and unlikely to be influenced by response bias related to country overrepresentation or differences in national physician workforce capacity.

## Discussion

In this study, we present survey findings on the real-life clinical practice strategies employed by physicians across continents and countries to timely and accurately diagnose KD.

The classification criteria for KD require the presence of at least four out of five clinical criteria and a fever ≥5 days [23], ensuring high specificity, especially for studies or registry enrolment. However, to support earlier recognition and prompt initiation of therapy, several guidelines now recommend diagnosis on the fourth day of fever when the clinical presentation is suggestive, or on the third day when assessed by experienced physicians [2, 3, 7, 24]. Other guidelines advise diagnosing KD in case of a fever for at least 4 days and in the presence of clinical signs and CALs [25], or have removed any minimum fever duration requirement [8]. Our survey revealed that approximately one-third of physicians diagnose complete KD after 4 days of fever if the clinical picture is clear, while a minority after 3 days. These findings suggest that the recently published guideline updates remain partially incorporated into daily practice. Notably, French physician reported diagnosing KD particularly early during disease course, which may reflect the influence of the recently published European and national guidelines [3, 24]. Our survey also showed that years of clinical experience did not significantly influence the likelihood of diagnosing complete KD before 5 days of fever.

Incomplete KD often presents with few or transient clinical signs, particularly in very young children [26], which can delay recognition and treatment [27], and has been reported to be associated with less frequent regression of CALs [17]. Some guidelines define incomplete KD as the presence of two to three clinical signs combined with systemic inflammation, supportive laboratory values, and the presence of CALs [2, 3, 24, 28, 29]. Other guidelines define incomplete KD in the absence of CALs when three to four clinical signs are present (which may include fever) [8], or permit diagnosis after ≥5 days of fever without requiring a minimum number of additional clinical features [25]. Our survey results indicate that nearly all respondents diagnose incomplete KD when a prolonged fever and three of the principal signs are present, despite the absence of CALs. Around half would diagnose KD with two signs, whereas only a few consider it when a single sign is present. These findings suggest that in real-life physicians are generally willing to make the diagnosis even in less clear cases, thereby ensuring early therapy. However, diagnostic confidence decreases as the number of symptoms declines. Fever remains an important feature for most physicians, with only a minority accepting afebrile presentations.

The presence of CALs is considered a specific criterion that supports a diagnosis of KD, particularly in patients who do not meet the full clinical criteria for a complete KD diagnosis [7]. Although CALs can also occur in other inflammatory conditions such as osteomyelitis, mycoplasmic pneumonia, viral infections and Still’s disease [30], *Z*-scores of 2.0–2.5 or above are more commonly observed in KD [2, 31]. CALs also occur in multisystem inflammatory syndrome in children (MIS-C), an important differential diagnosis for KD; however, they are typically less prevalent and milder [32]. Distinct clinical and laboratory features, including Vβ23.1-expressing T cells in MIS-C, help to differentiate between the two conditions [33, 34]. Due to the high specificity of CALs in KD, guidelines generally recommend diagnosing incomplete KD when CALs are present, even if fewer clinical criteria are met. This includes patients with three clinical signs (including fever) [8] and those with suspected KD despite normal laboratory values [2, 3, 24]. Our survey revealed that nearly half of respondents would diagnose KD in a child with ≥5 days of fever and CALs, even in the absence of other signs, indicating the strong diagnostic weight of this finding. This approach was consistent across countries. On the other hand, CALs are usually not an early manifestation of the disease and they do not develop in all patients [26, 35, 36], which is a diagnostic challenge when symptoms are few or incomplete, particularly at disease onset.

To improve diagnostic accuracy in cases of suspected incomplete KD, the guidelines incorporate additional features, including laboratory signs and cardiovascular findings, as well as other supportive clinical signs [3, 7, 8]. In our survey, respondents not only identified these additional factors, but also graded their importance in real-life practice. They revealed that inflammation markers, particularly elevated CRP, ESR, and thrombocytosis after day 7, are very important factors, particularly in distinguishing KD from viral infections [37, 38]. Furthermore, a strong reduction in well-being, a recognized feature of KD [39], was considered moderately important by a substantial proportion of respondents and may help differentiate KD from viral or localized bacterial infections [39, 40]. Young age was also frequently cited as a factor supporting diagnosis, reflecting clinicians’ awareness that infants often present with oligosymptomatic disease while carrying a high-risk of CALs [26]. Overall, respondents reported using a combination of laboratory tests and echocardiographic findings to distinguish KD from other conditions, which illustrates the complexity of real-world diagnostic decision-making. These findings indicate that physicians use multiple supportive features beyond formal diagnostic criteria when evaluating suspected incomplete KD.

The identification of patients at high-risk for developing CALs remains difficult, and decisions regarding intensified initial treatment are often unclear in daily practice. Early recognition is crucial, since timely, intensified therapy can significantly reduce cardiac complications [16, 27]. Nevertheless, even low-risk patients may benefit from short-term treatment with glucocorticoids or other immunomodulators, given that there is still a small risk of permanent arterial injury in this patient group [8]. Although risk scores exist (Kobayashi, Egami or Sano) [8], their inconsistent performance limits their widespread application. Consequently, different national guidelines recommend varying criteria for treatment intensification to prevent CALs development (Table 2) [3, 7, 8, 24, 25, 41–43]. In our survey, age <1 year was the most frequently cited risk factor for CALs. In addition to a *Z*-score >2, physicians also take other early occurring echocardiographic signs into account when assessing risk, such as ventricular dysfunction and signs of pericarditis and myocarditis. More than half of respondents rated nearly all proposed risk factors (except ‘other chronic disease’) as at least moderately important, indicating a reliance on broad clinical judgement rather than strict scoring tools.

**Table 2:** Comparison of potential risk factors for CAAs development used for intensified treatment across different guidelines.

Guidelines	Risk factors
SHARE (Europe) [3]	Kobayashi score ≥5, <1 year of age, pathological laboratory findings (↓serum sodium, ↑ALT and AST, hypoalbuminemia, ↑CRP, ↓platelet count, ↓haemoglobin), HLH, shock, CAA
AHA (USA) [2, 7]	Baseline CAA *Z*-score ≥2.5, infants ≤6 months or in high-risk category using the Sano risk score
Japan [8]	Kobayashi score ≥5, Egami score ≥3, Sano score ≥2
France [22]	*Severe disease:* <1 year of age; shock, MAS, CAA/dilatation *Risk for cardiac sequelae:* IVIG initiation after 10th day of fever, IVIG resistance, incomplete KD, <1 year of age; male, severe inflammation (leucocytosis, thrombocytosis, anaemia, high CRP)
Italy [23]	<1 year of age, ↑CRP, ↑ALT, AST level, hypoalbuminemia, severe anaemia at disease onset, CAA, MAS or KD shock syndrome
Germany [39]	*Recommended:* initial Z-score >2, ≤1 year of age, severe disease (e.g. MAS, shock) *Considered:* ≥7 years of age, male, pathological laboratory findings (↑inflammatory markers, ↑ALT and AST, hypoalbuminemia, anaemia, ↓serum sodium), initiation of therapy ≤4th or >14th day of illness
Sweden [40]	Early anomalies in coronary arteries, <1 year of age with severe disease, development of/at risk for MAS
Spain [41]	*Severe disease:* shock, <1 year of age + 2 high-risk factors for IVIG resistance, CAA or other cardiac pathology *High-risk:* <1 year of age, CRP ≥90 mg/l, ESR ≥80 mm/h, thrombocytosis ≥900 000/mm^3^, hepatic dysfunction, albumin ≤2.5 g/dl, plasma sodium ≤133 mEq/l, Hb at least 2 g/dl under age limit

AHA, American Heart Association; ALT, alanine aminotransferase; AST, aspartate aminotransferase; CAA, coronary artery aneurysm; Hb, haemoglobin, HLH, haemophagocytic lymphohistiocytosis; KD, Kawasaki disease; MAS, macrophage activation syndrome; SHARE initiative, Single Hub and Access point for paediatric Rheumatology in Europe; ↑, raised; ↓, low.

Our study has several limitations. About one-third of initial participants did not complete the entire questionnaire, revealing a non-response bias, which is a common issue in survey research. Missing responses were more frequent in Brazil, Ghana and New Zealand, which participated in the pilot phase. Responses may be subject to social desirability bias, where respondents may report guideline-consistent rather than actual behaviour. Question misinterpretation, centre-specific practice patterns or limited experience should also be considered. Broader global participation would strengthen representativeness. The uneven distribution of response rates across countries makes direct comparisons challenging. Several subgroup analyses were based on small sample sizes, making them underpowered and potentially missing significant differences. Given the exploratory nature of this study and the number of comparisons made, there is a potential for inflated Type I error, and *P-*value should be interpreted cautiously.

Furthermore, while incorporating real-life clinical practice into guideline development may be valuable, it requires careful consideration. Lower diagnostic thresholds increase sensitivity at the expense of specificity, with potential consequences for diagnostic accuracy and treatment decisions, particularly in the absence of a disease-specific biomarker. Further prospective studies are therefore needed to evaluate the impact of such approaches on clinical outcomes.

In conclusion, this international survey highlights the real-life clinical strategies employed for diagnosing KD and identifying patients at risk of CALs. Although clinical strategies and assessments of disease presentation were largely consistent, relevant country-specific differences were observed that do not appear to be influenced by years of clinical experience. Complete KD is generally recognized with ≥5 days of fever and four clinical criteria, but a substantial minority, particularly in Europe and France, make diagnoses earlier. Incomplete KD is most often identified with fever and three criteria, though many clinicians assign decisive diagnostic weight to CALs, even in the absence of other criteria. Infant age and elevated coronary *Z*-scores are the factors most commonly used to identify patients at high-risk for coronary involvement. Integrating these real-life practices into future studies and subsequent guideline development may contribute to improved disease management and patient outcomes.

## Supplementary Material

keag340_Supplementary_Data

## Data Availability

The data are available from the corresponding author upon reasonable request.
