# Supplementary material for: Diagnostic approaches to Kawasaki disease worldwide: the results from the JIR-CliPS network
Source: Rheumatology (Oxford). 2026 Jun 26;65(7):keag340. doi: 10.1093/rheumatology/keag340 (PMC13378453; doi:10.1093/rheumatology/keag340)
Supplement: keag340_Supplementary_Data [file keag340_supplementary_data.zip › Supplementary data S3_weighted.docx]

A complete KD can be diagnosed in children with 4 out of 5 clinical criteria and fever:

|  | **Total**  **(n=192)** | **Weighted analysis by doctors’ capacity per country* (n=192)** |
| --- | --- | --- |
| **≥5 days of fever** | 130 (67.7%) | 67.6% |
| **≥4 days of fever** | 31 (16.1%) | 17.5% |
| **≥3 days of fever** | 26 (13.5%) | 13.1% |
| **≥2 days of fever** | 1 (0.5%) | 0.1% |
| **≥1 day of fever** | 1 (0.5%) | 0.4% |
| **5 clinical criteria and no history of fever** | 3 (1.6%) | 0.9% |

**Weighted χ² = 18.20, p = 0.574*

An incomplete KD can be diagnosed in children with:

|  | **Options** | **Total** | **Weighted analysis by doctors’ capacity per country** |
| --- | --- | --- | --- |
| **Scenario 1***  **(n=178/189)** | Fever ≥5 days and 3 out of 5 clinical criteria | 84 (47.2%) | 45.3% |
|  | Fever ≥5 days and 2 out of 5 clinical criteria | 73 (41.0%) | 41.7% |
|  | Fever ≥5 days and 1 out of 5 clinical criteria | 21 (11.8%) | 13.1% |
| Scenario 2**  (n=31/189) | No fever, 4 out of 5 clinical criteria | 22 (71.0%) | 70.2% |
|  | No fever, 3 out of 5 clinical criteria | 6 (19.4%) | 20.3% |
|  | No fever, 2 out of 5 clinical criteria | 3 (9.7%) | 9.6% |
|  | No fever, 1 out of 5 clinical criteria | 0 (0%) | 0% |
| Scenario 3***  (n=147/189) | Fever ≥5 days, CALs, 3 out of 5 clinical criteria | 11 (7.5%) | 5.9% |
|  | Fever ≥5 days, CALs, 2 out of 5 clinical criteria | 30 (20.4%) | 23.2% |
|  | Fever ≥5 days, CALs, 1 out of 5 clinical criteria | 22 (15%) | 10.5% |
|  | Fever ≥5 days, CALs, 0 out of 5 clinical criteria | 84 (57.1%) | 60.4% |

**Weighted χ² = 10.08, p = 0.259*

***Weighted χ² = 5.44, p = 0.710*

****Weighted χ² = 11.46, p = 0.490*
