# Supplementary material for: Diagnostic approaches to Kawasaki disease worldwide: the results from the JIR-CliPS network
Source: Rheumatology (Oxford). 2026 Jun 26;65(7):keag340. doi: 10.1093/rheumatology/keag340 (PMC13378453; doi:10.1093/rheumatology/keag340)
Supplement: keag340_Supplementary_Data [file keag340_supplementary_data.zip › Supplementary data S2_sensitivity.docx]

A COMPLETE KD can be diagnosed in children with 4 out of 5 clinical criteria AND FEVER:

|  | **Total**  **(n=192)** | **Without 5 most-represented countries**  **(n=110)** | **Without France**  **(n=168)** | **Without Turkey**  **(n=171)** | **Without Brazil**  **(n=178)** | **Without Spain**  **(n=179)** | **Without Germany**  **(n=182)** |
| --- | --- | --- | --- | --- | --- | --- | --- |
| **≥5 days of fever** | 130 (67.7%) | 90 (72.6%) | 122 (72.6%) | 115 (67.3%) | 118 (66.3%) | 118 (65.9%) | 125 (68.7%) |
| **≥4 days of fever** | 31 (16.1%) | 16 (12.9%) | 23 (13.7%) | 28 (16.4%) | 31 (17.4%) | 30 (16.8%) | 28 (15.4%) |
| **≥3 days of fever** | 26 (13.5%) | 14 (11.3%) | 19 (11.3%) | 23 (13.5%) | 24 (13.5%) | 26 (14.5%) | 24 (13.2%) |
| **≥2 days of fever** | 1 (0.5%) | 1 (0.8%) | 1 (0.6%) | 1 (0.6%) | 1 (0.6%) | 1 (0.6%) | 1 (0.5%) |
| **≥1 day of fever** | 1 (0.5%) | 1 (0.8%) | 1 (0.6%) | 1 (0.6%) | 1 (0.6%) | 1 (0.6%) | 1 (0.5%) |
| **5 clinical criteria and no history of fever** | 3 (1.6%) | 2 (1.6%) | 2 (1.2%) | 3 (1.8%) | 3 (1.7%) | 3 (1.7%) | 3 (1.6%) |

AN INCOMPLETE KD can be diagnosed in children with:

|  | **Total**  **(n=189)** | **Without 5 most-represented countries**  **(n=123)** | **Without France**  **(n=167)** | **Without Turkey**  **(n=168)** | **Without Brazil**  **(n=175)** | **Without Spain**  **(n=176)** | **Without Germany**  **(n=179)** |
| --- | --- | --- | --- | --- | --- | --- | --- |
| **Fever ≥5 days and 3 out of 5 clinical criteria** | 178 (94.2%) | 144 (92.7%) | 156 (93.4%) | 159 (94.6%) | 164 (93.7%) | 165 (93.8%) | 168 (93.9%) |
| **Fever ≥5 days and 2 out of 5 clinical criteria** | 94 (49.7%) | 55 (44.7%) | 81 (48.5%) | 81 (48.2%) | 87 (49.7%) | 87 (49.4%) | 88 (49.2%) |
| **Fever ≥5 days and 1 out of 5 clinical criteria** | 21 (11.1%) | 12 (9.8%) | 19 (11.4%) | 16 (9.5%) | 21 (12%) | 21 (11.9%) | 19 (10.6%) |
| No fever, 4 out of 5 clinical criteria | 31 (16.4%) | 20 (16.3%) | 28 (16.8%) | 30 (17.9%) | 31 (17.7%) | 29 (16.5%) | 26 (14.5%) |
| No fever, 3 out of 5 clinical criteria | 9 (4.8%) | 7 (5.7%) | 9 (5.4%) | 8 (4.8%) | 9 (5.1%) | 9 (5.1%) | 8 (4.5%) |
| No fever, 2 out of 5 clinical criteria | 3 (1.6%) | 2 (1.6%) | 3 (1.8%) | 2 (1.2%) | 3 (1.7%) | 3 (1.7%) | 3 (1.7%) |
| No fever, 1 out of 5 clinical criteria | 0 (0%) | 0 (0%) | 0 (0%) | 0 (0%) | 0 (0%) | 0 (0%) | 0 (0%) |
| Fever ≥5 days, CALs, 3 out of 5 clinical criteria | 147 (77.8%) | 94 (76.4%) | 129 (77.2%) | 129 (76.8%) | 140 (80.0%) | 140 (79.5%) | 137 (76.5%) |
| Fever ≥5 days, CALs, 2 out of 5 clinical criteria | 136 (72.0%) | 85 (69.1%) | 118 (70.7%) | 120 (71.4%) | 129 (73.7%) | 129 (73.3%) | 126 (70.4%) |
| Fever ≥5 days, CALs, 1 out of 5 clinical criteria | 106 (56.1%) | 67 (54.5%) | 92 (55.1%) | 93 (55.4%) | 101 (57.7%) | 103 (58.5%) | 97 (54.2%) |
| Fever ≥5 days, CALs, 0 out of 5 clinical criteria | 84 (44.4%) | 50 (40.7%) | 71 (42.5%) | 72 (42.9%) | 80 (45.7%) | 82 (46.6%) | 77 (43.0%) |
