# Supplementary material for: Diagnostic approaches to Kawasaki disease worldwide: the results from the JIR-CliPS network
Source: Rheumatology (Oxford). 2026 Jun 26;65(7):keag340. doi: 10.1093/rheumatology/keag340 (PMC13378453; doi:10.1093/rheumatology/keag340)
Supplement: keag340_Supplementary_Data [file keag340_supplementary_data.zip › Supplementary figure S1.pptx]

## Slide 1
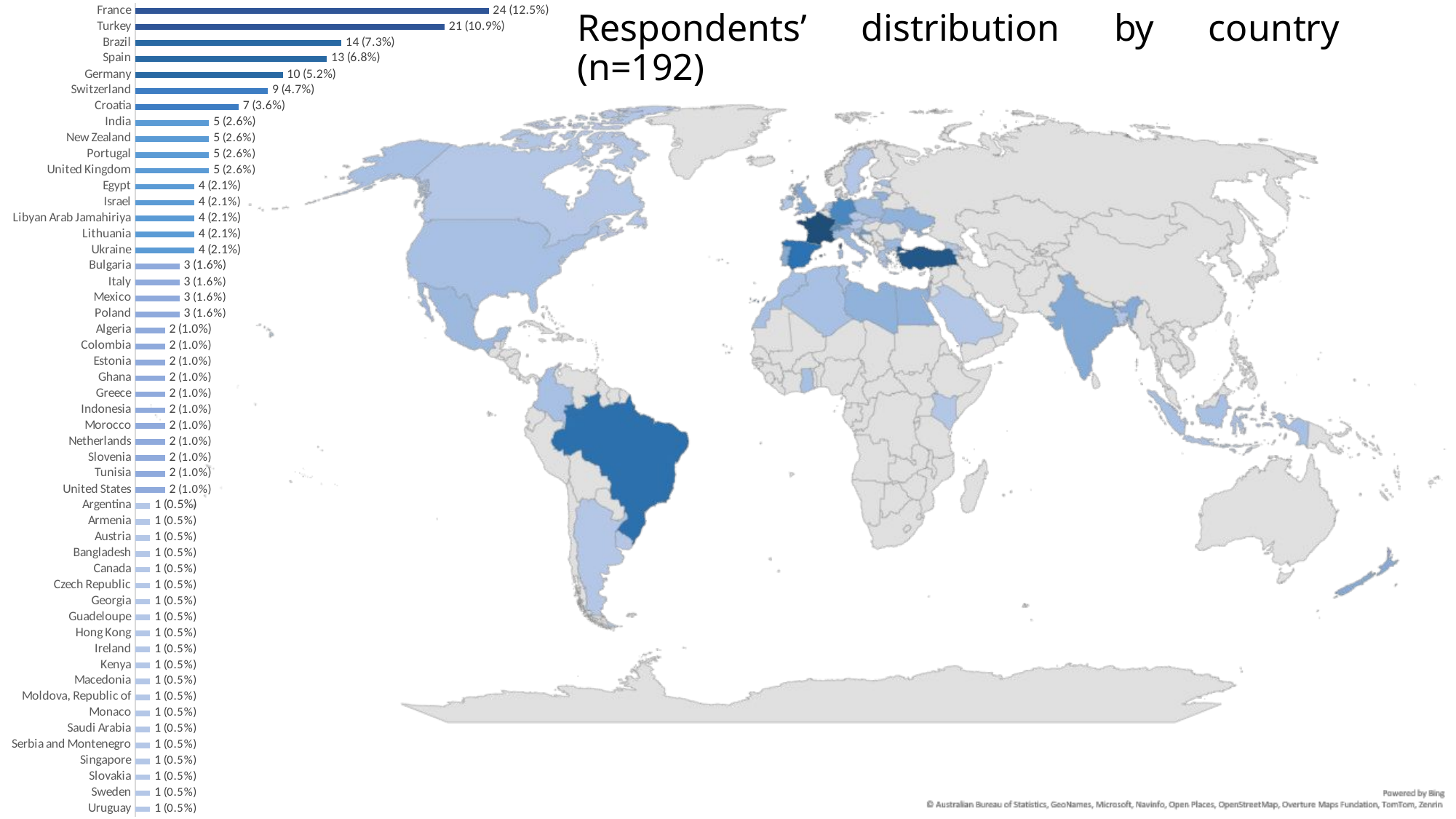

### Chart
| Category | |
|---|---|
| France | 24.0 |
| Turkey | 21.0 |
| Brazil | 14.0 |
| Spain | 13.0 |
| Germany | 10.0 |
| Switzerland | 9.0 |
| Croatia | 7.0 |
| India | 5.0 |
| New Zealand | 5.0 |
| Portugal | 5.0 |
| United Kingdom | 5.0 |
| Egypt | 4.0 |
| Israel | 4.0 |
| Libyan Arab Jamahiriya | 4.0 |
| Lithuania | 4.0 |
| Ukraine | 4.0 |
| Bulgaria | 3.0 |
| Italy | 3.0 |
| Mexico | 3.0 |
| Poland | 3.0 |
| Algeria | 2.0 |
| Colombia | 2.0 |
| Estonia | 2.0 |
| Ghana | 2.0 |
| Greece | 2.0 |
| Indonesia | 2.0 |
| Morocco | 2.0 |
| Netherlands | 2.0 |
| Slovenia | 2.0 |
| Tunisia | 2.0 |
| United States | 2.0 |
| Argentina | 1.0 |
| Armenia | 1.0 |
| Austria | 1.0 |
| Bangladesh | 1.0 |
| Canada | 1.0 |
| Czech Republic | 1.0 |
| Georgia | 1.0 |
| Guadeloupe | 1.0 |
| Hong Kong | 1.0 |
| Ireland | 1.0 |
| Kenya | 1.0 |
| Macedonia | 1.0 |
| Moldova, Republic of | 1.0 |
| Monaco | 1.0 |
| Saudi Arabia | 1.0 |
| Serbia and Montenegro | 1.0 |
| Singapore | 1.0 |
| Slovakia | 1.0 |
| Sweden | 1.0 |
| Uruguay | 1.0 |# Respondents’ distribution by country (n=192)
