# Supplementary figures and images for: Diagnostic approaches to Kawasaki disease worldwide: the results from the JIR-CliPS network
Source: Rheumatology (Oxford). 2026 Jun 26;65(7):keag340. doi: 10.1093/rheumatology/keag340 (PMC13378453; doi:10.1093/rheumatology/keag340)

# Diagnostic approaches to Kawasaki disease (KD)

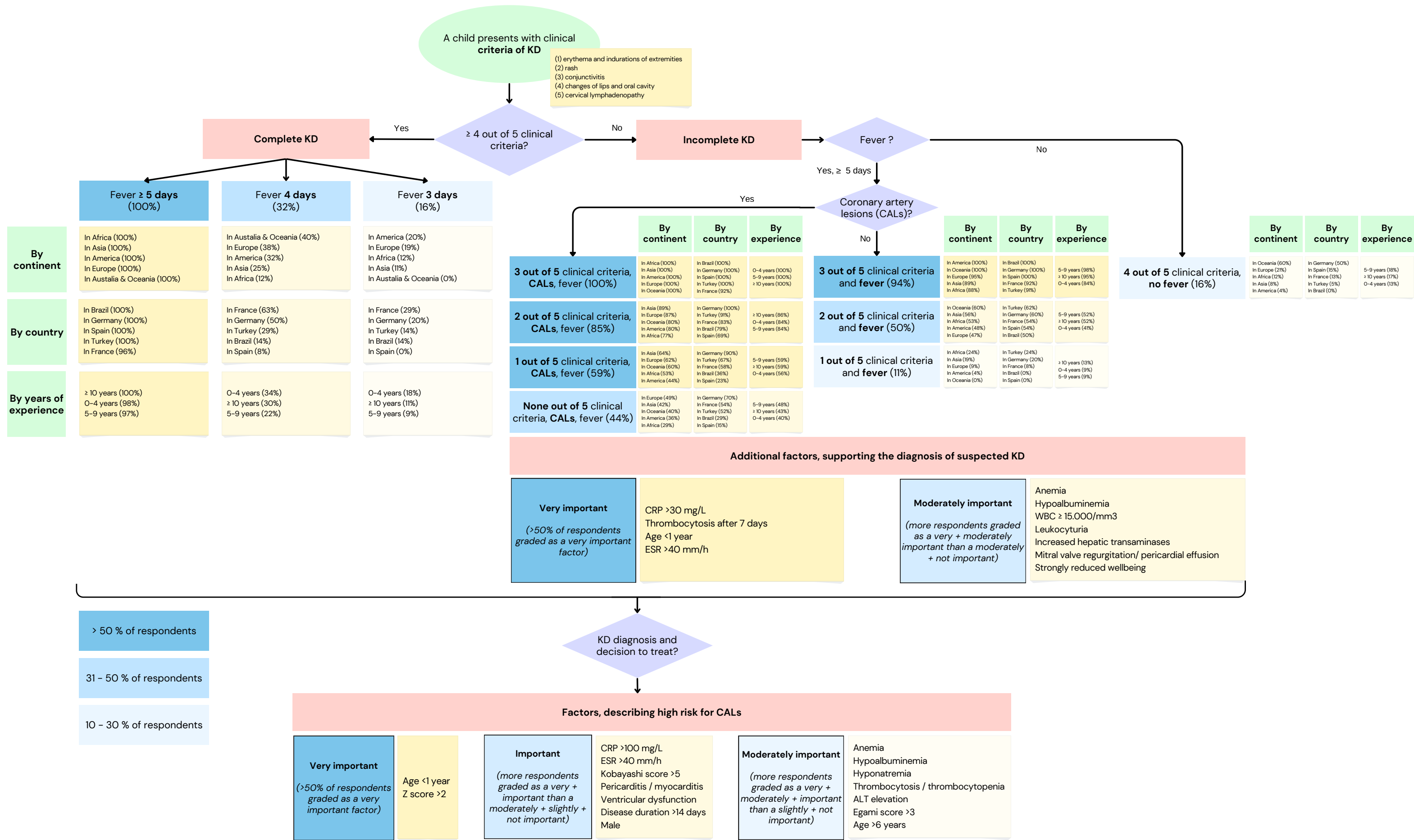

Supplement: keag340_Supplementary_Data [file keag340_supplementary_data.zip › Supplementary figure S2.pdf]

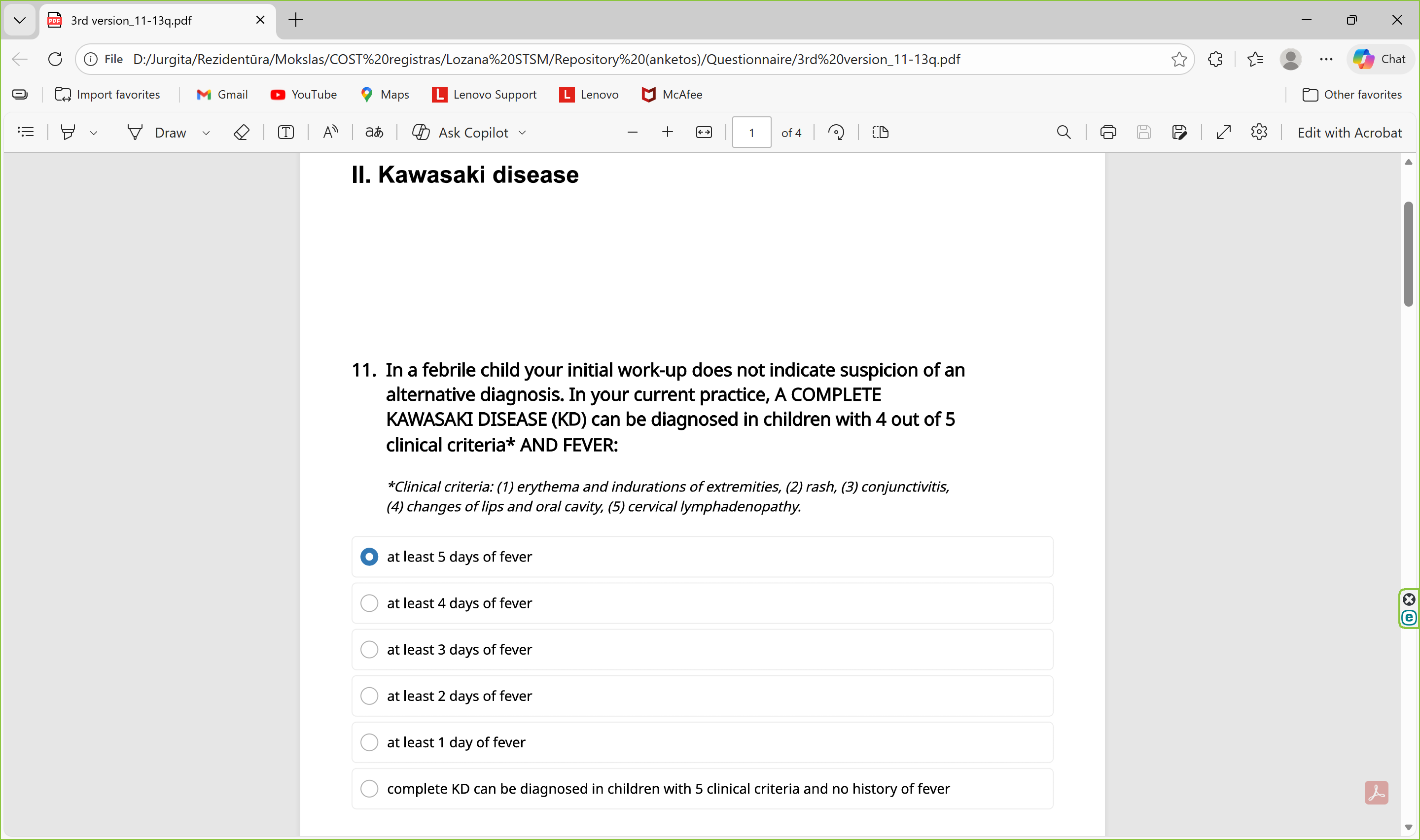


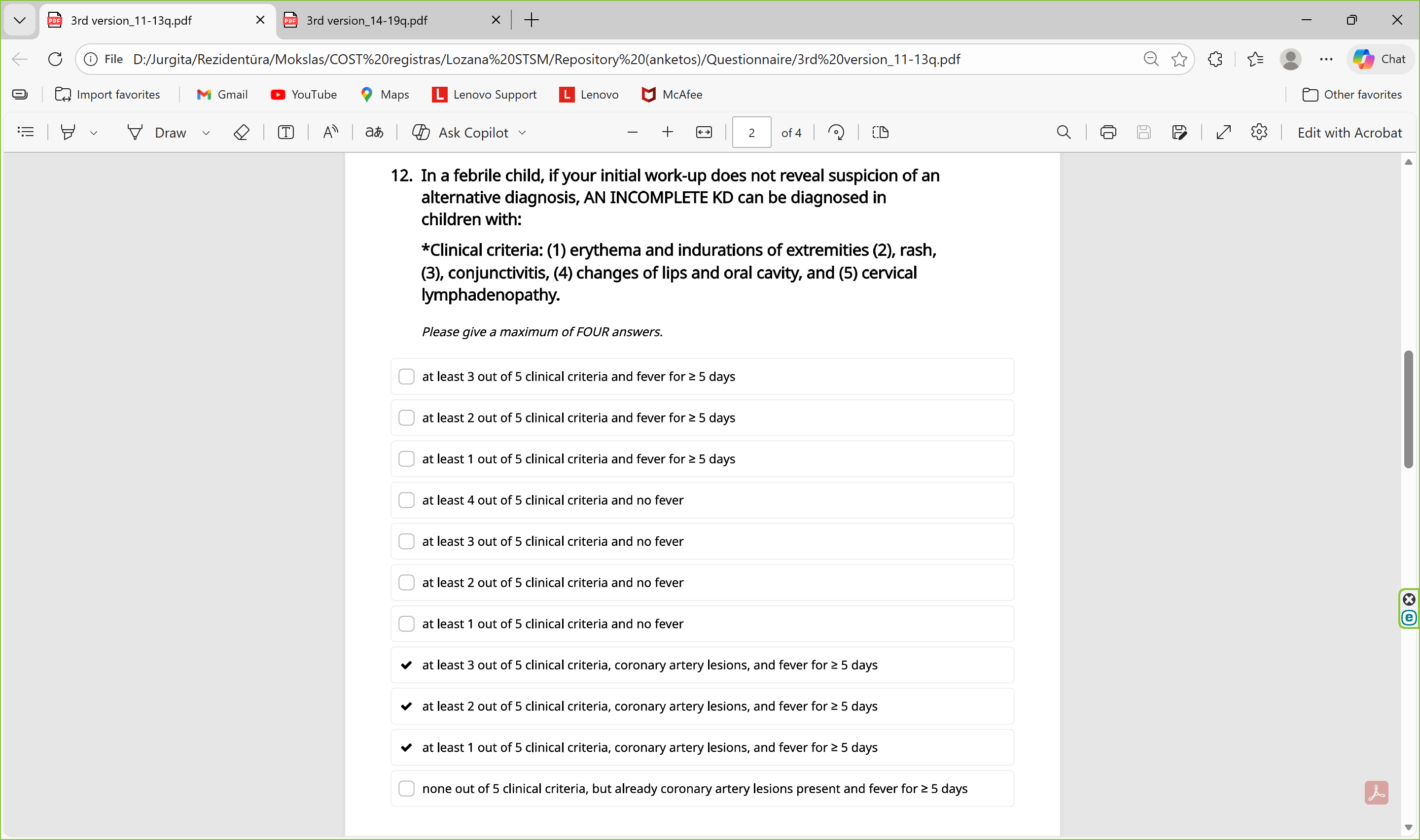


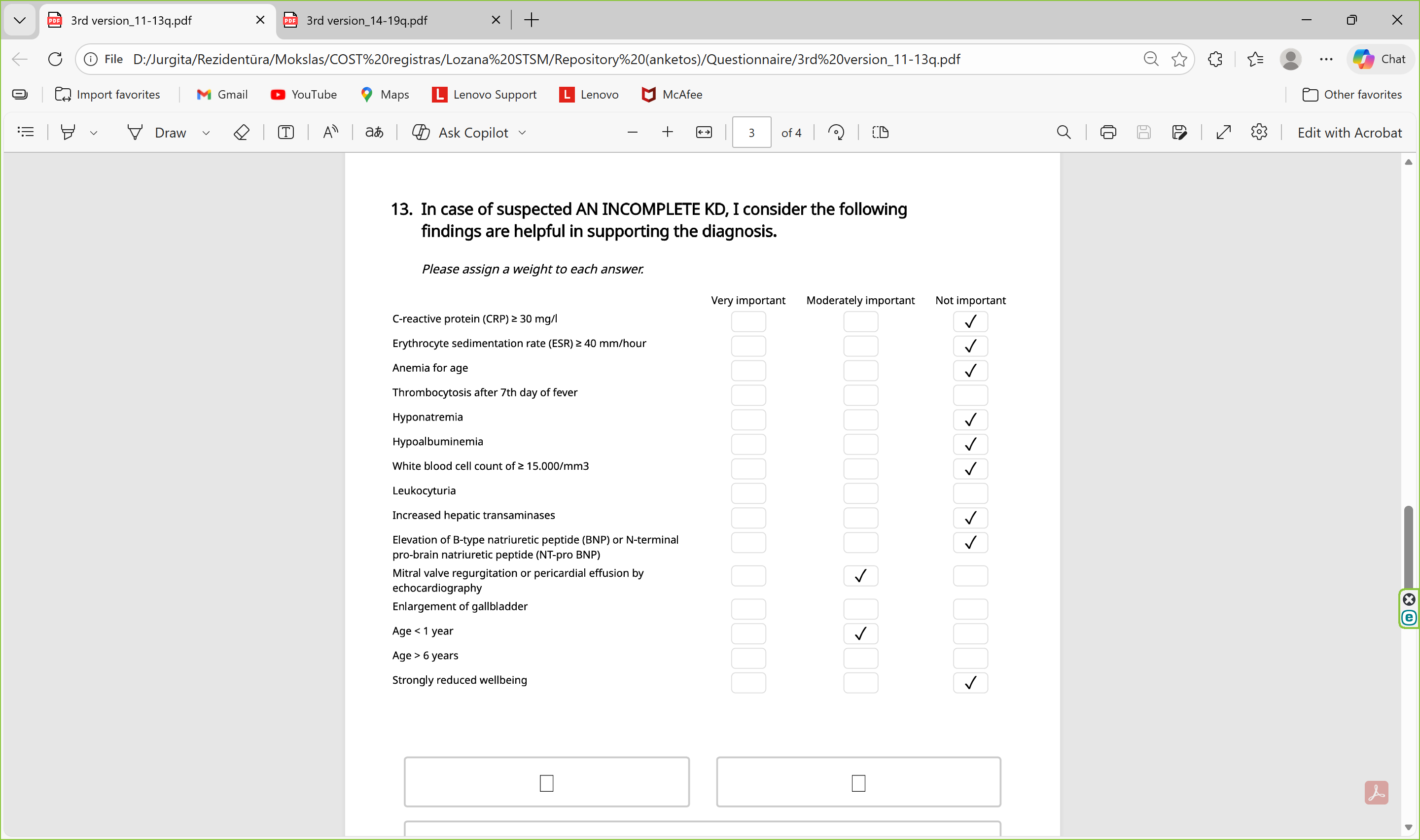


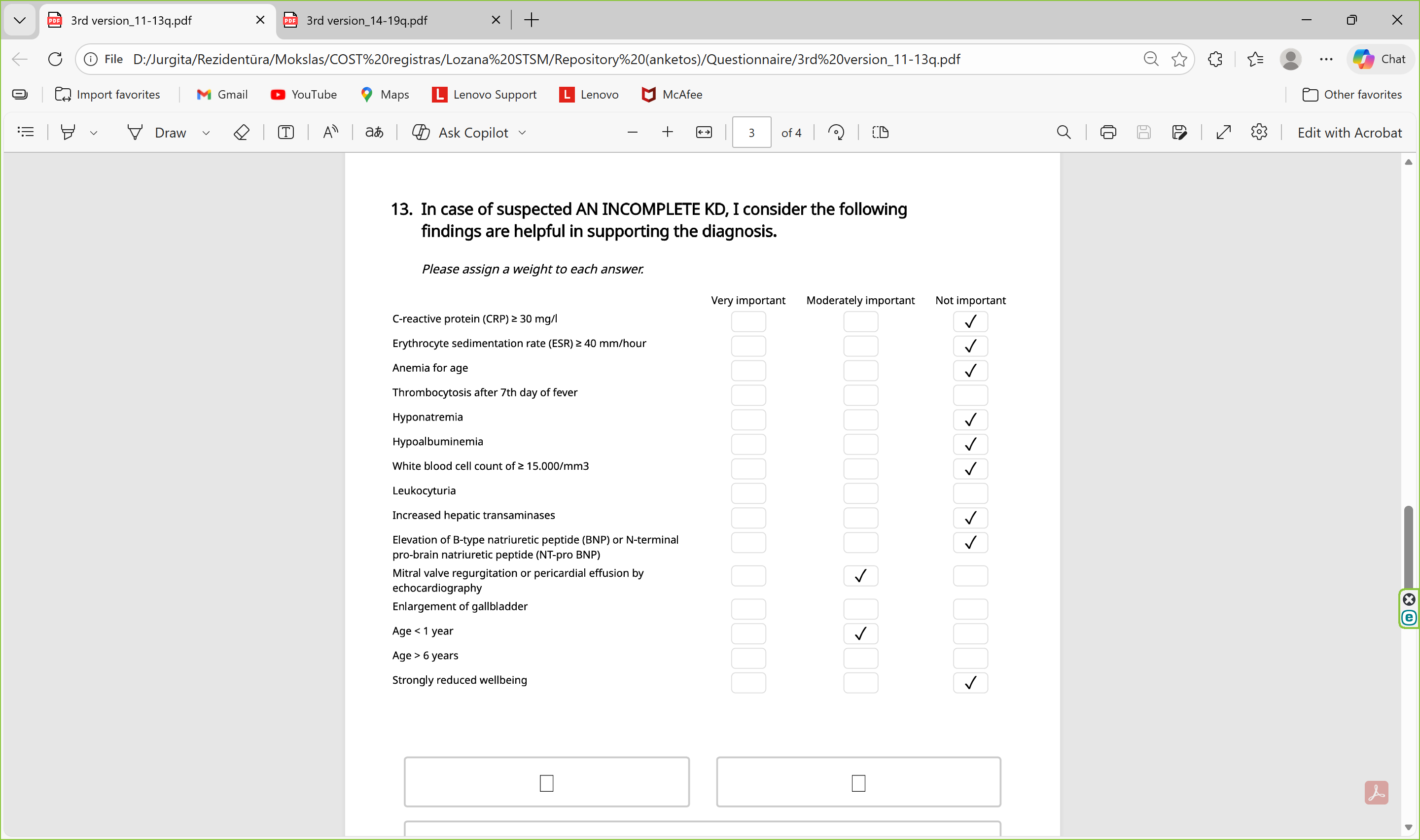

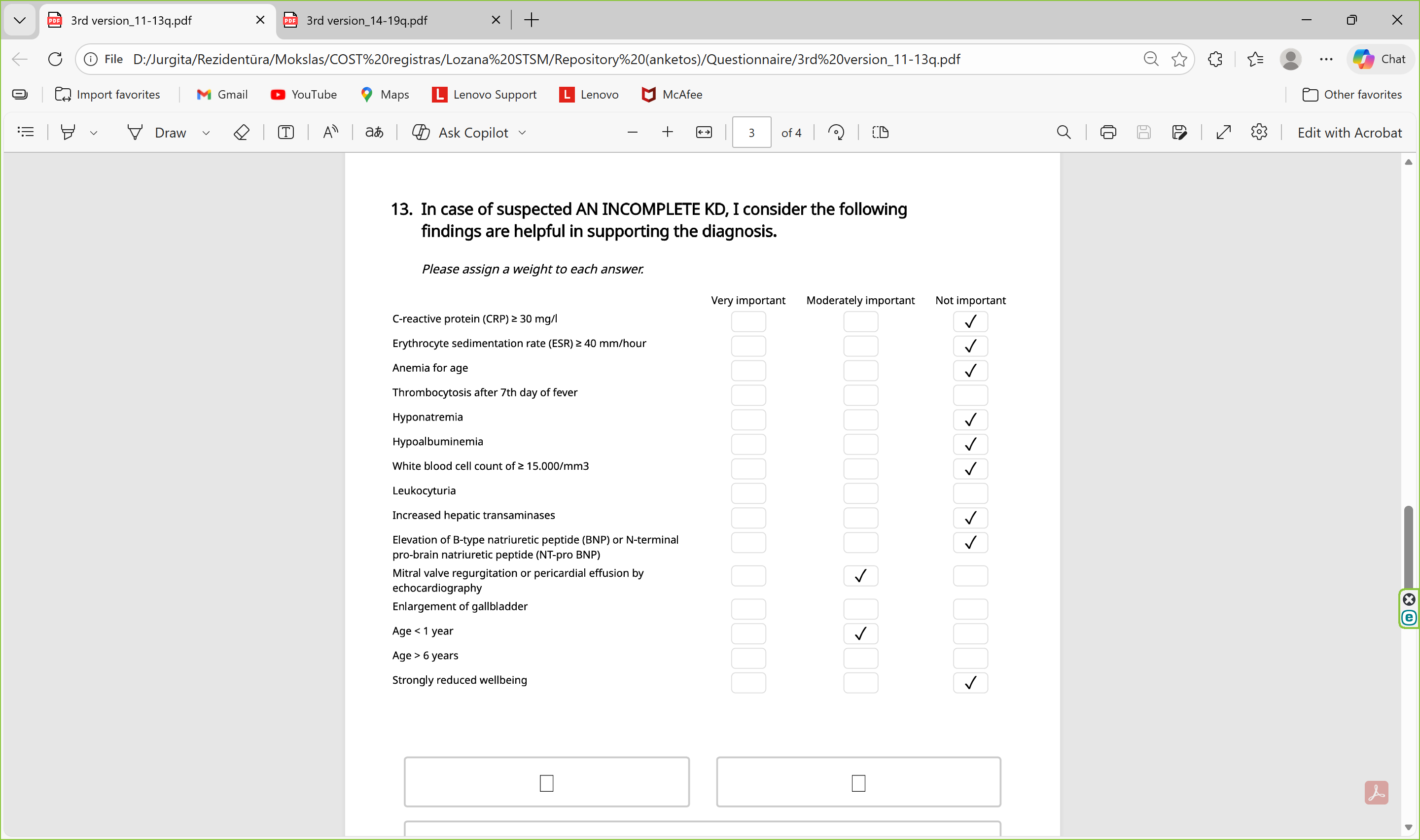

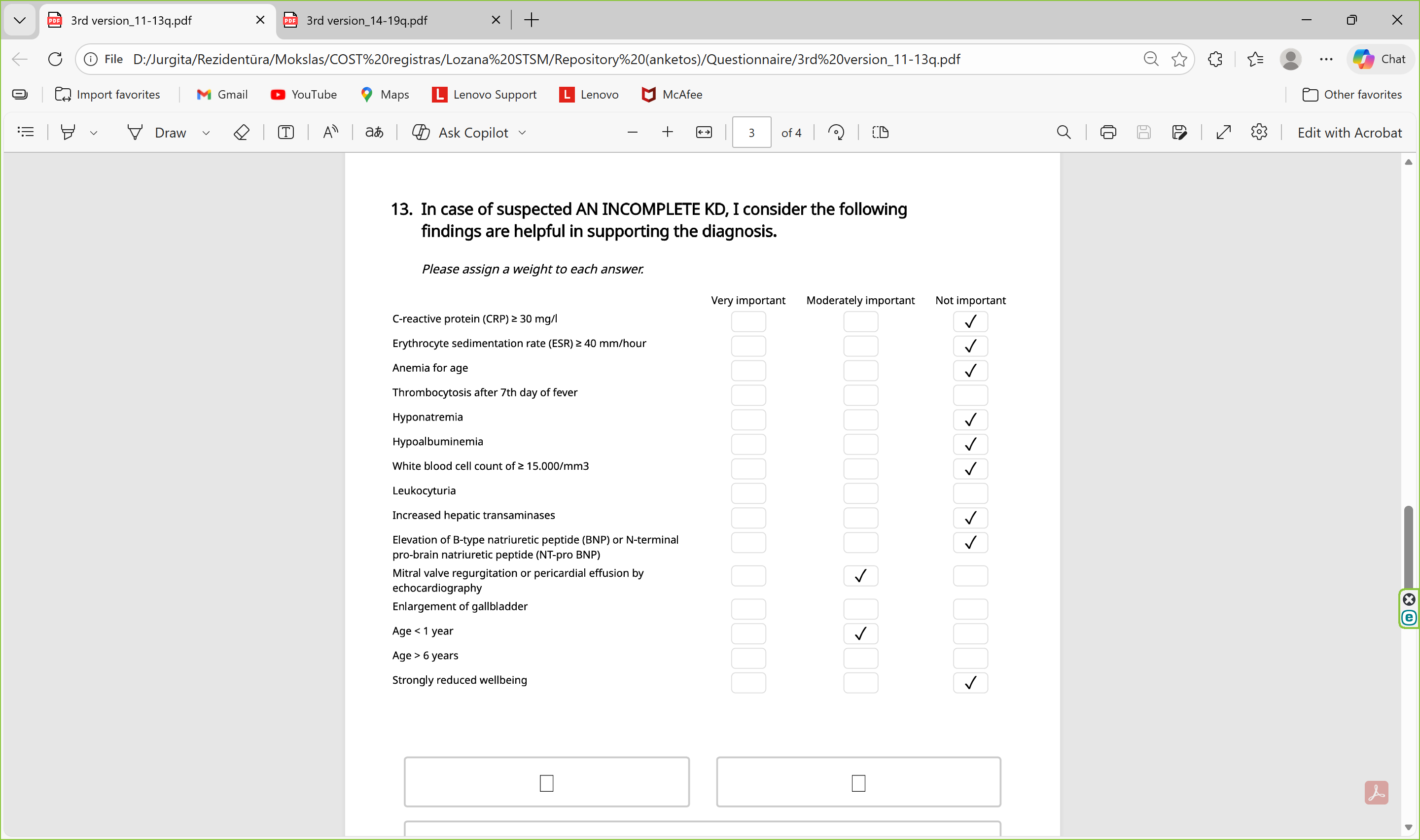


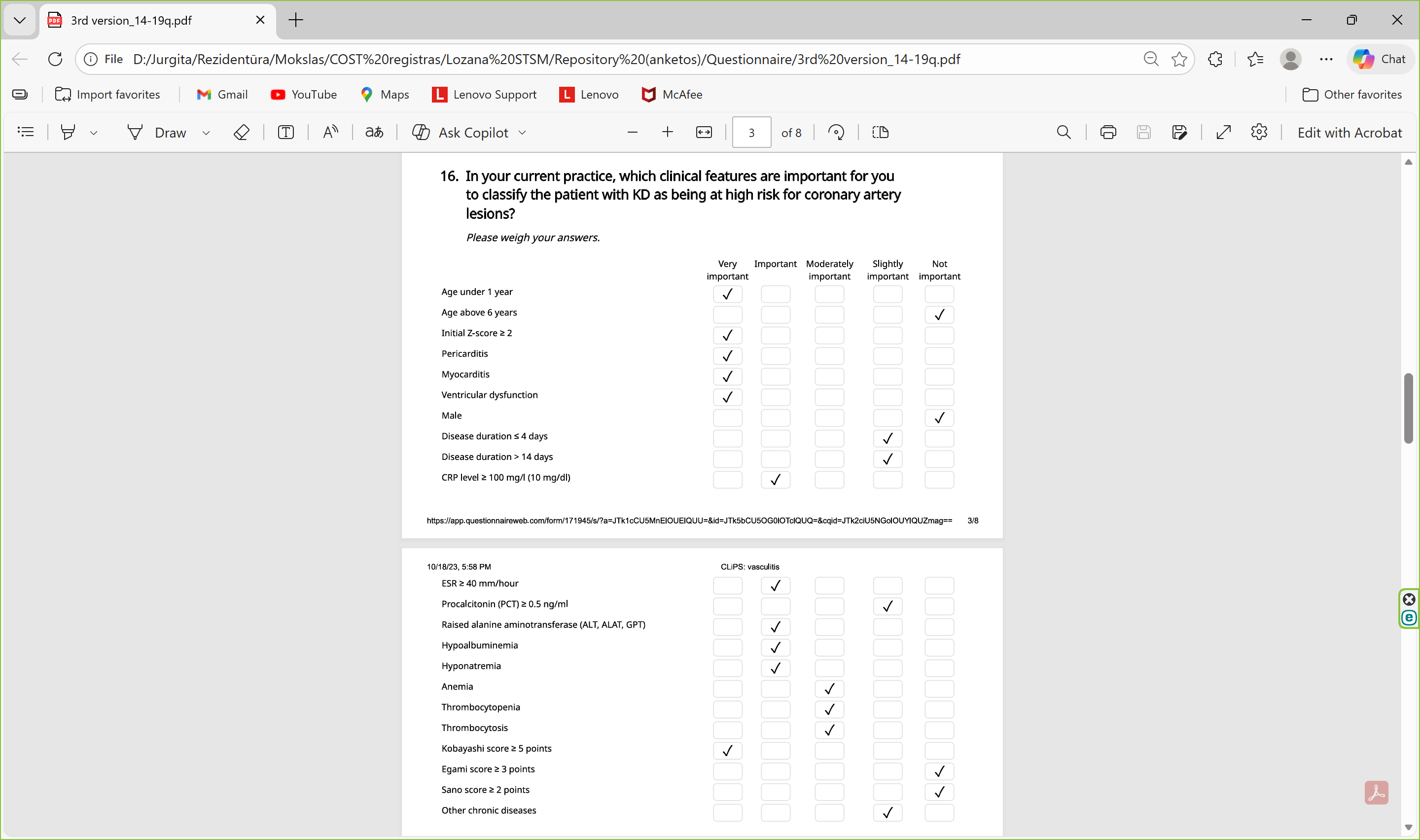


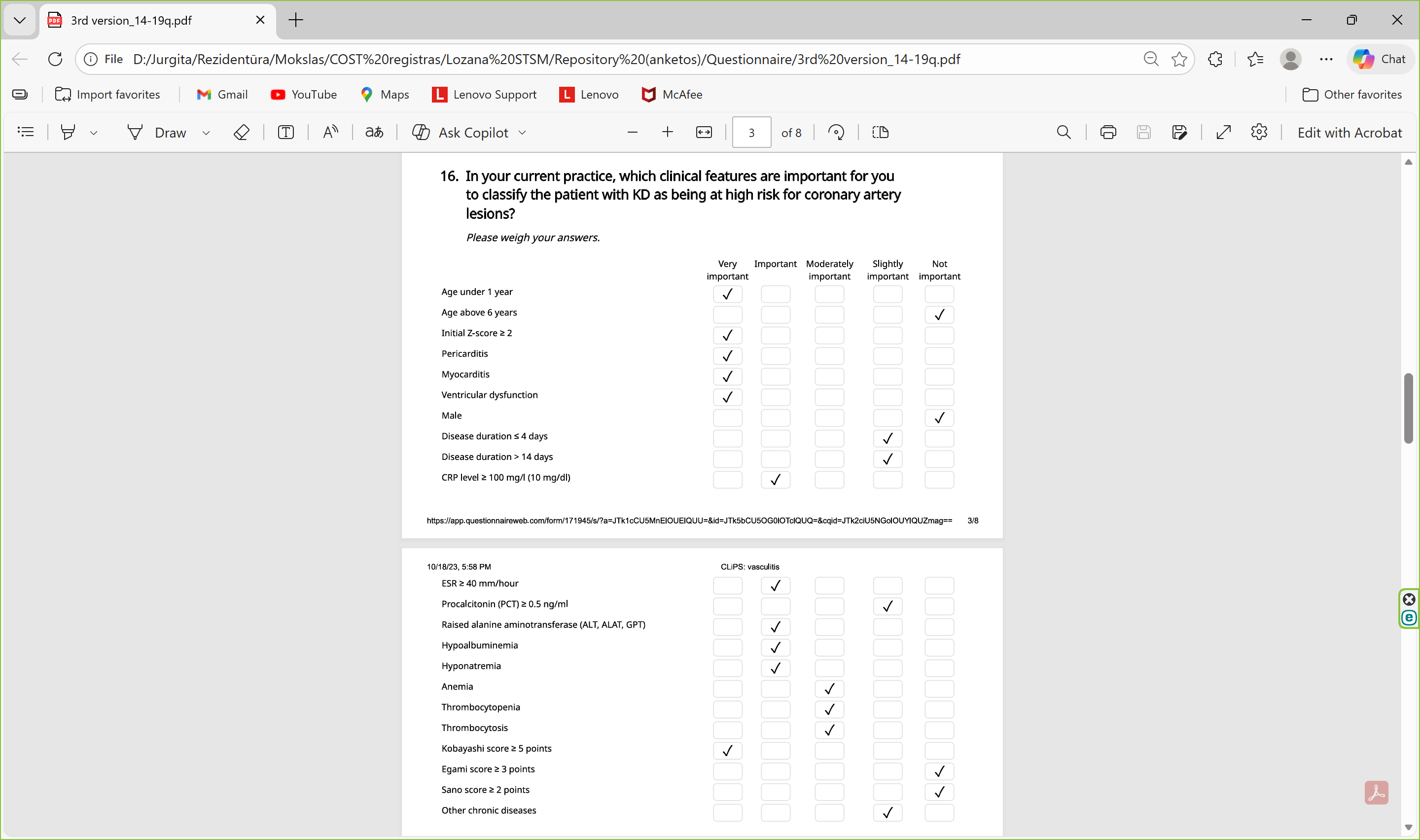

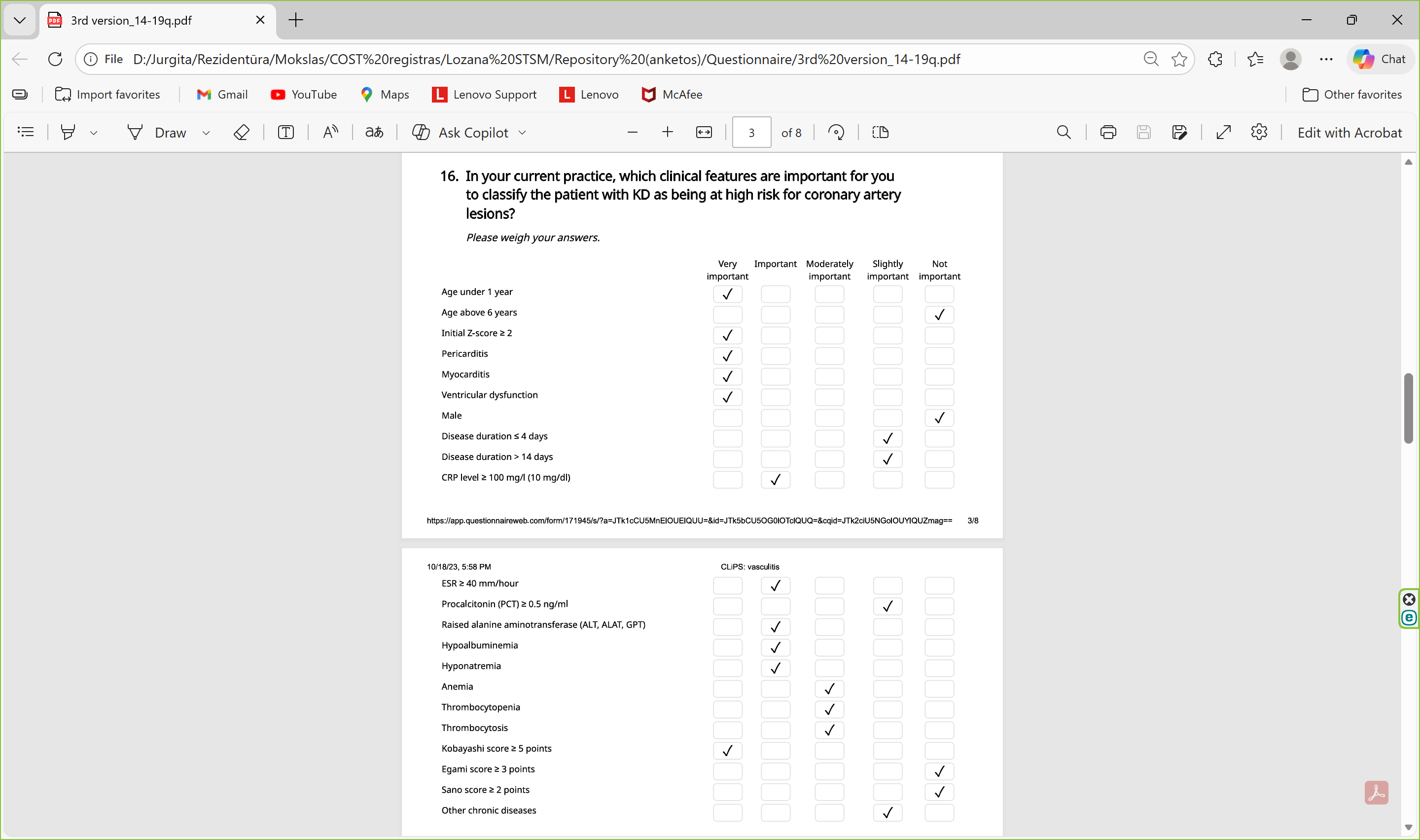

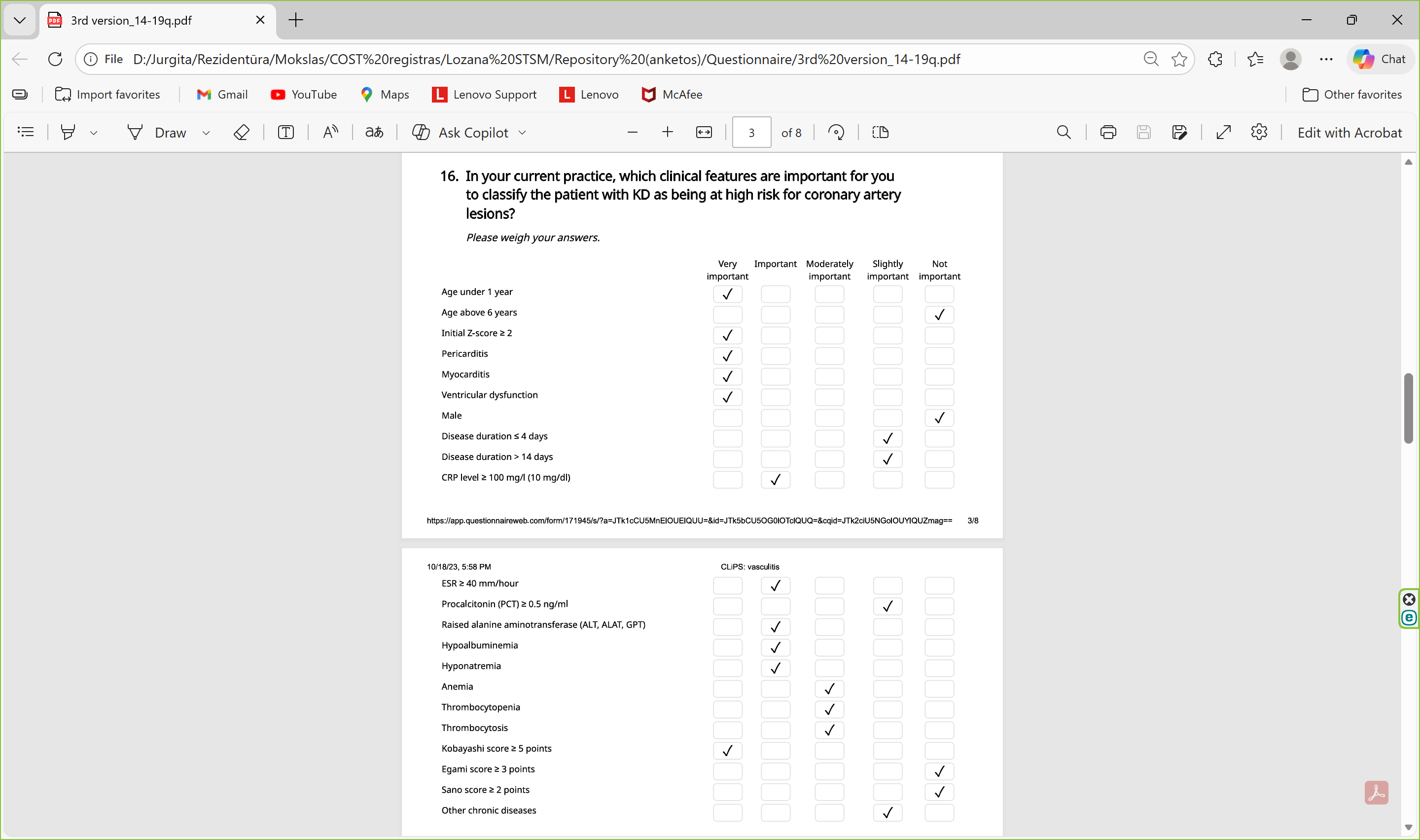

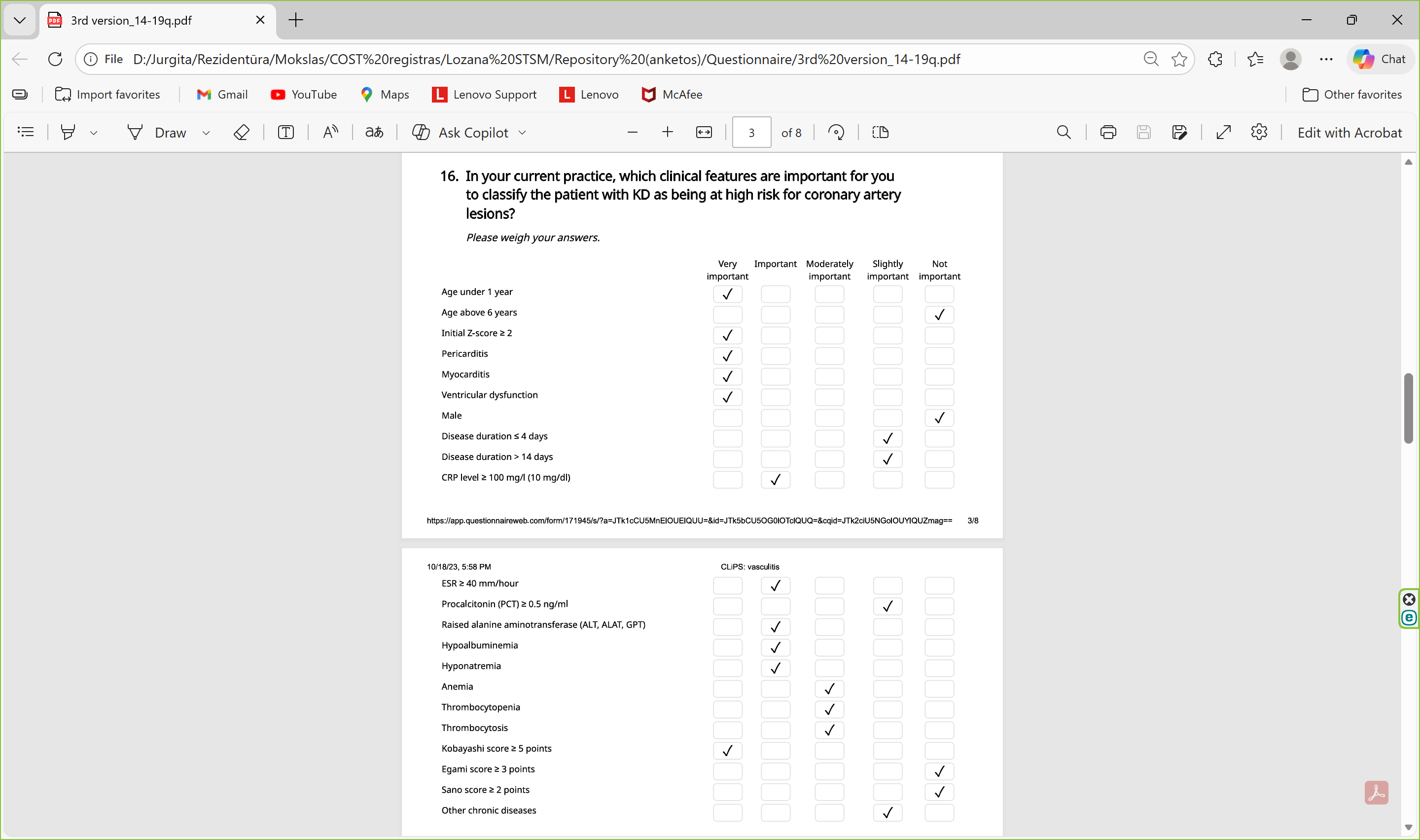

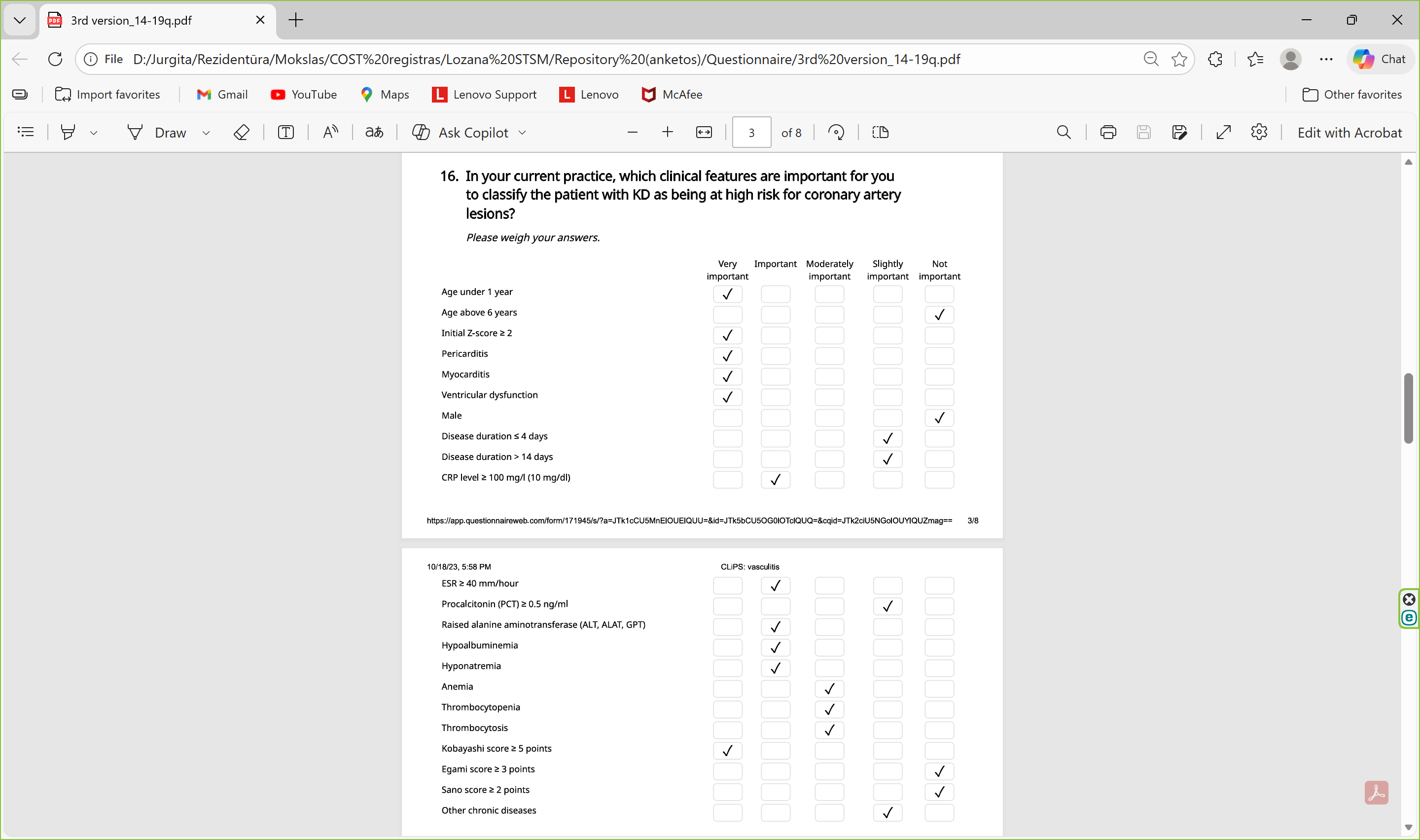

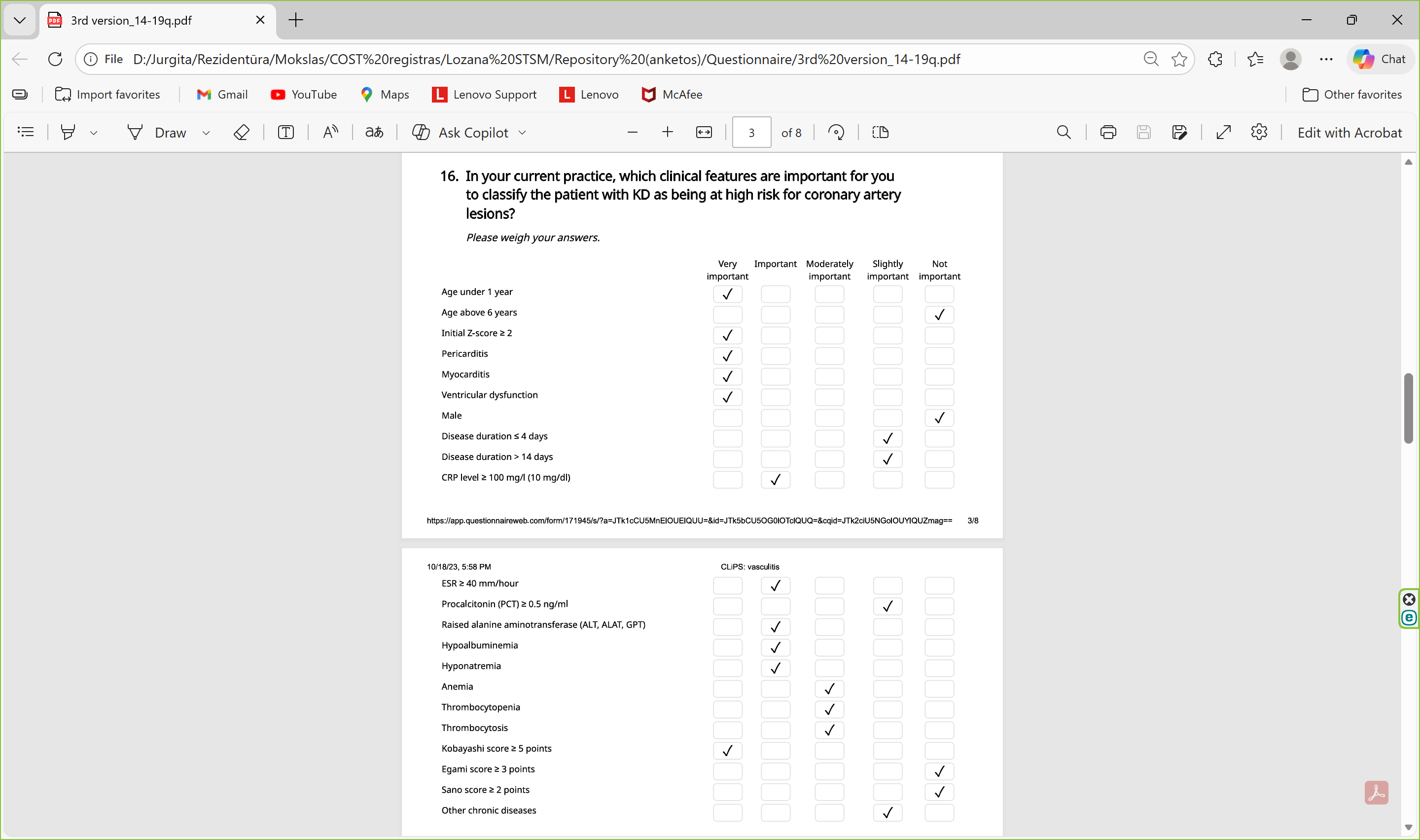

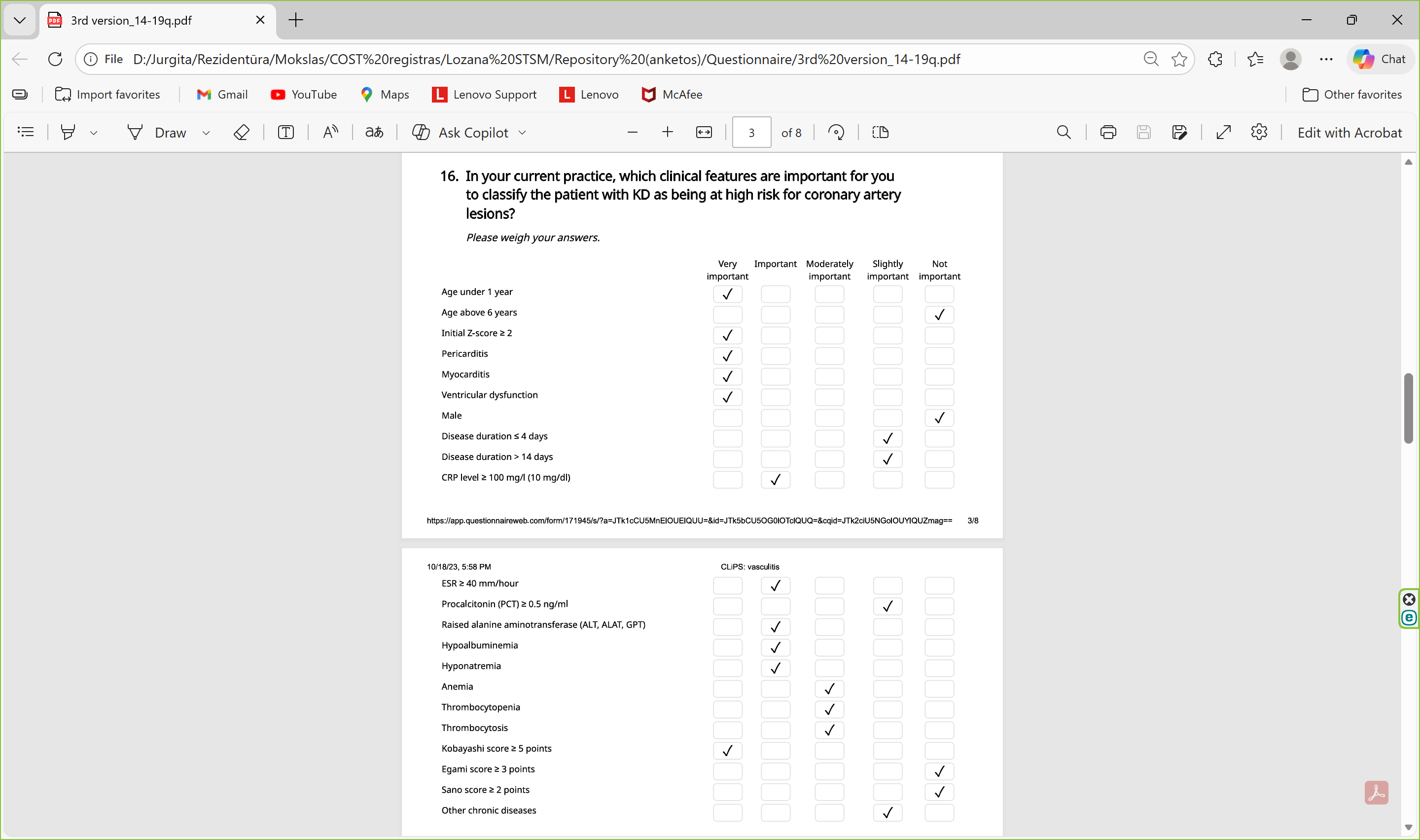

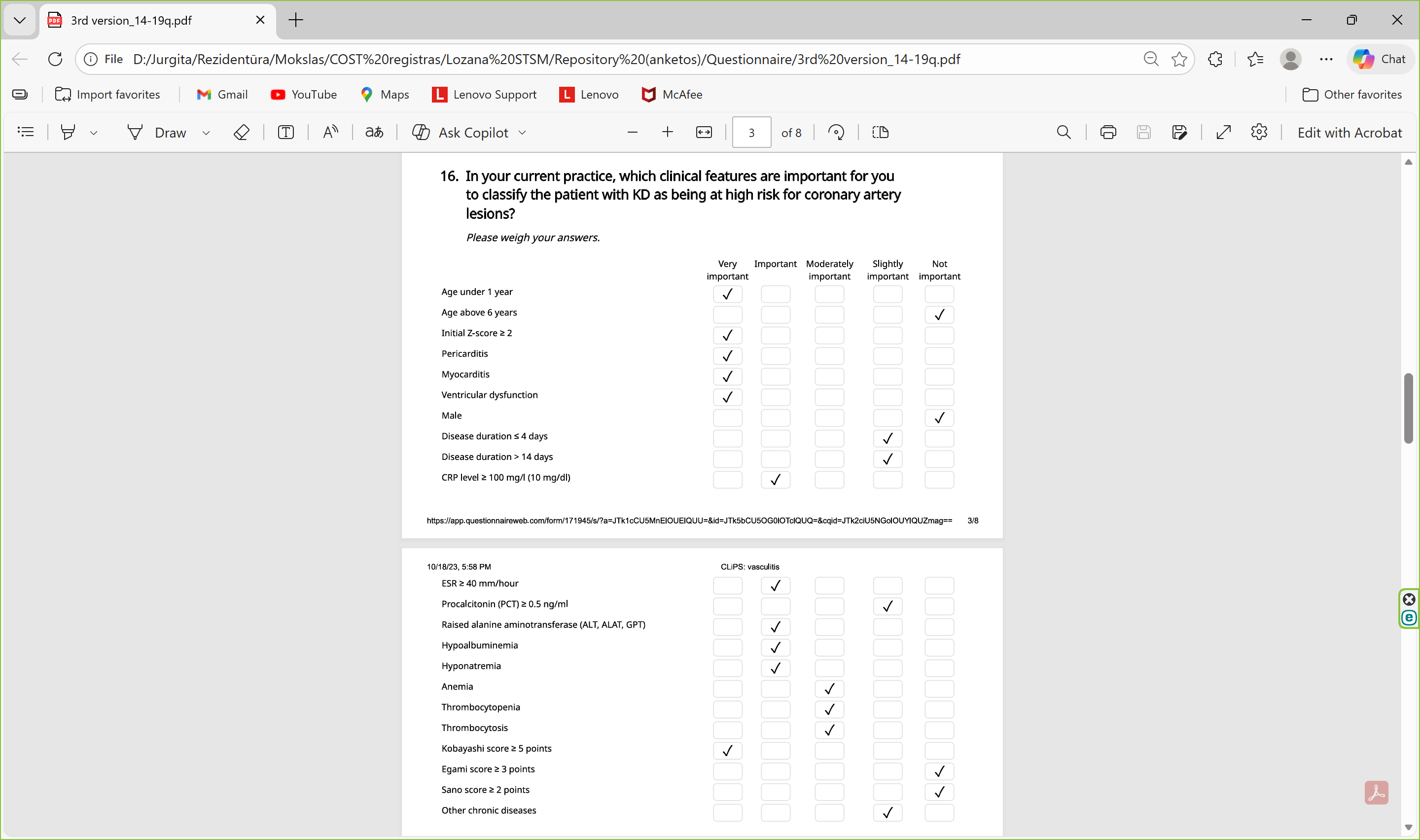

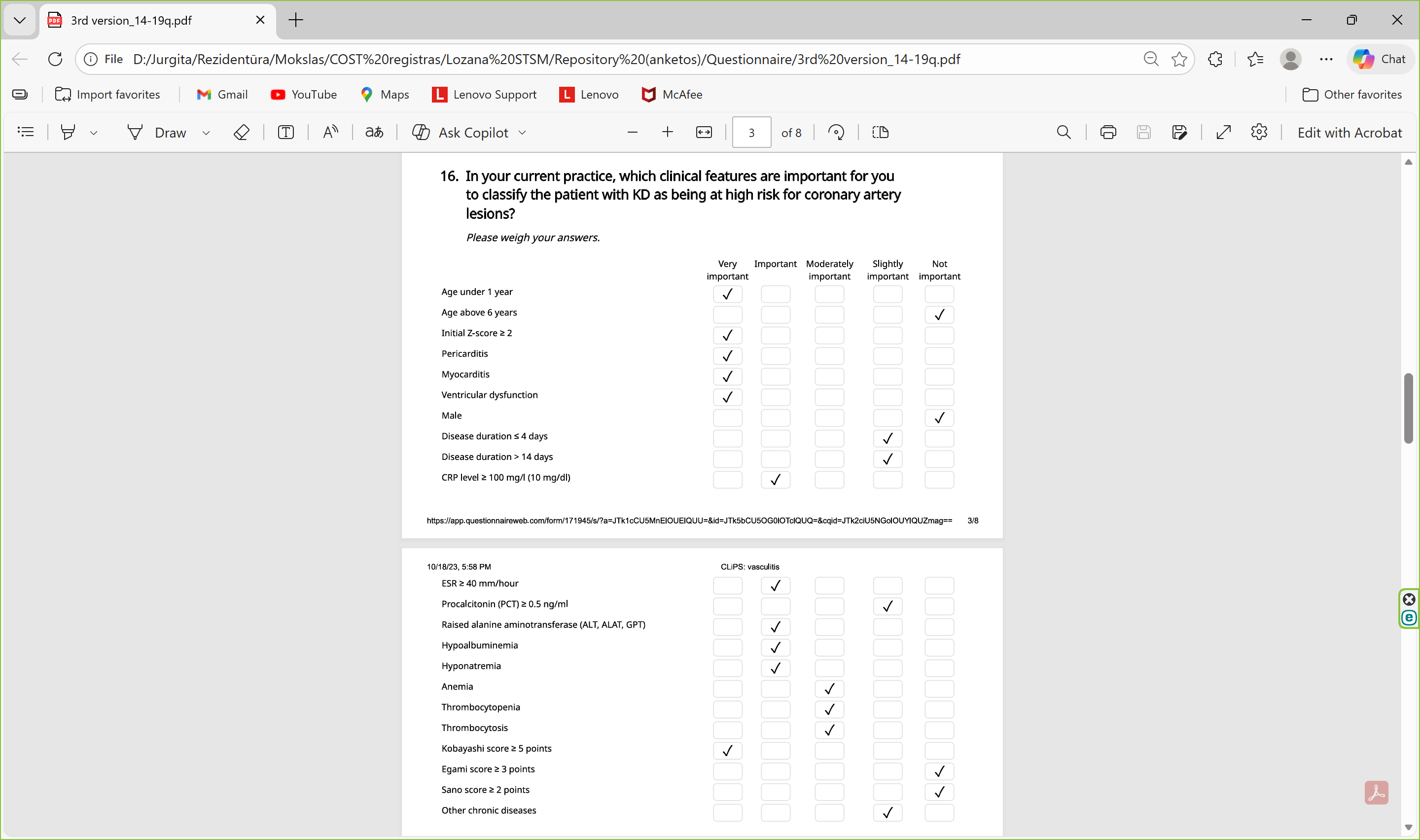

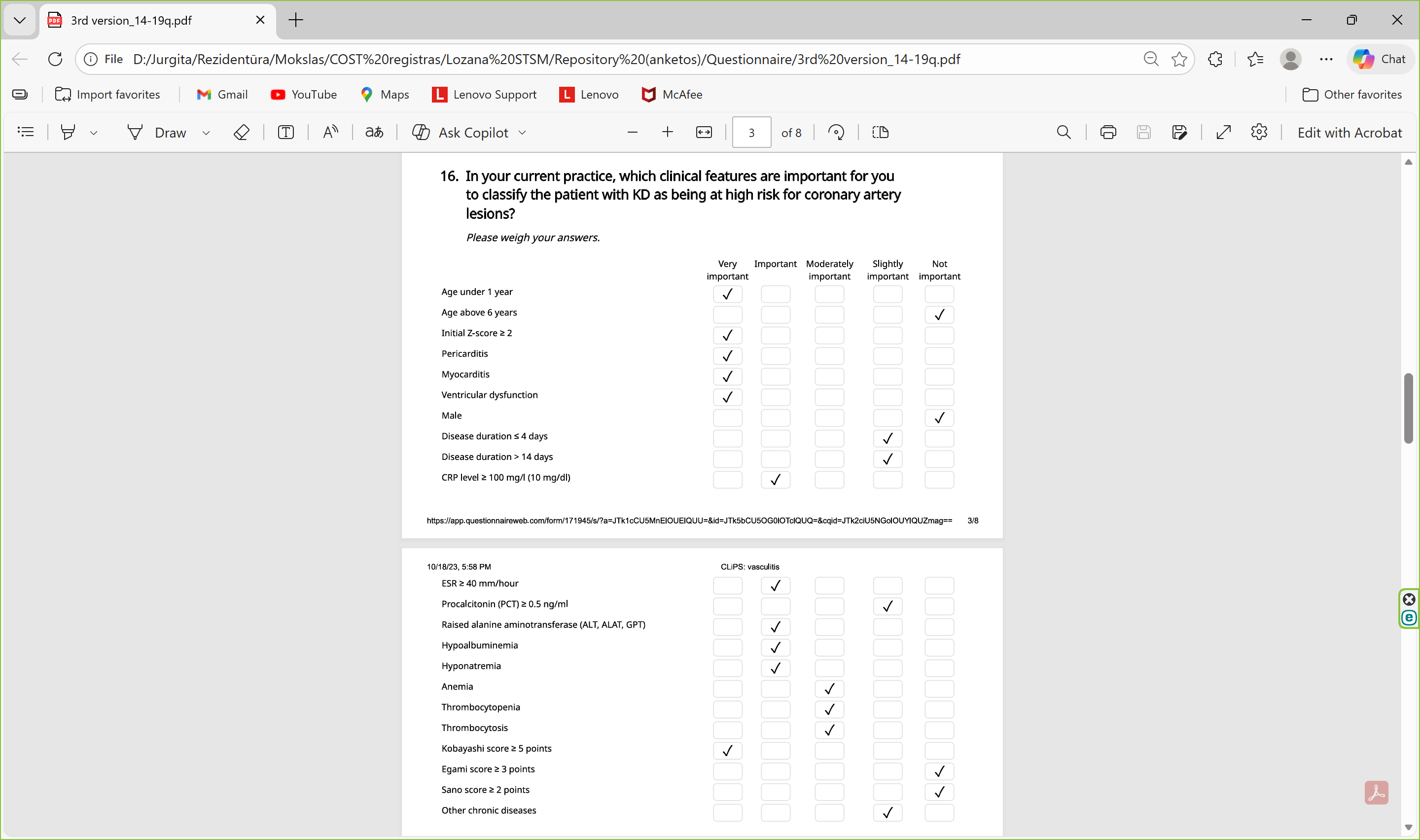

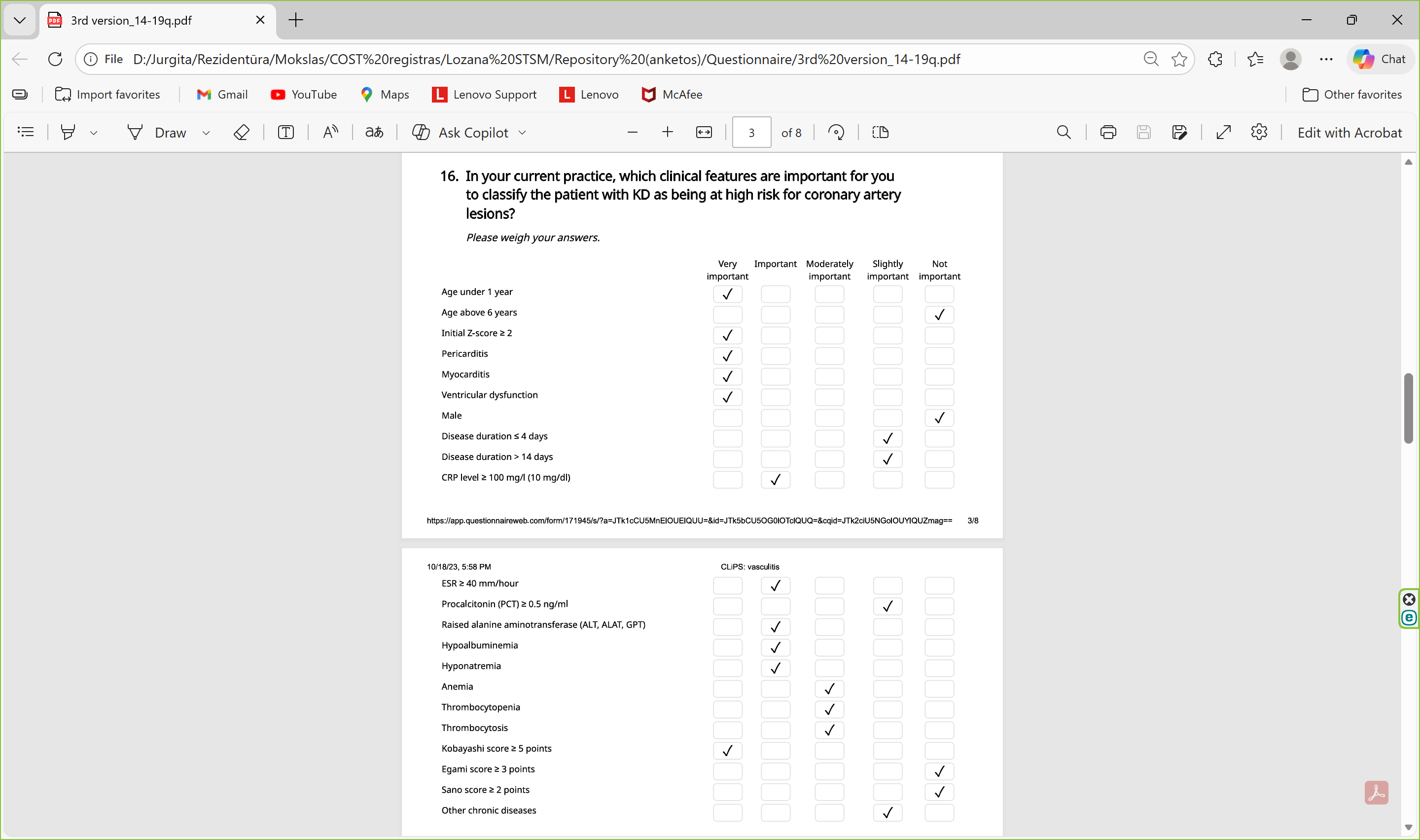


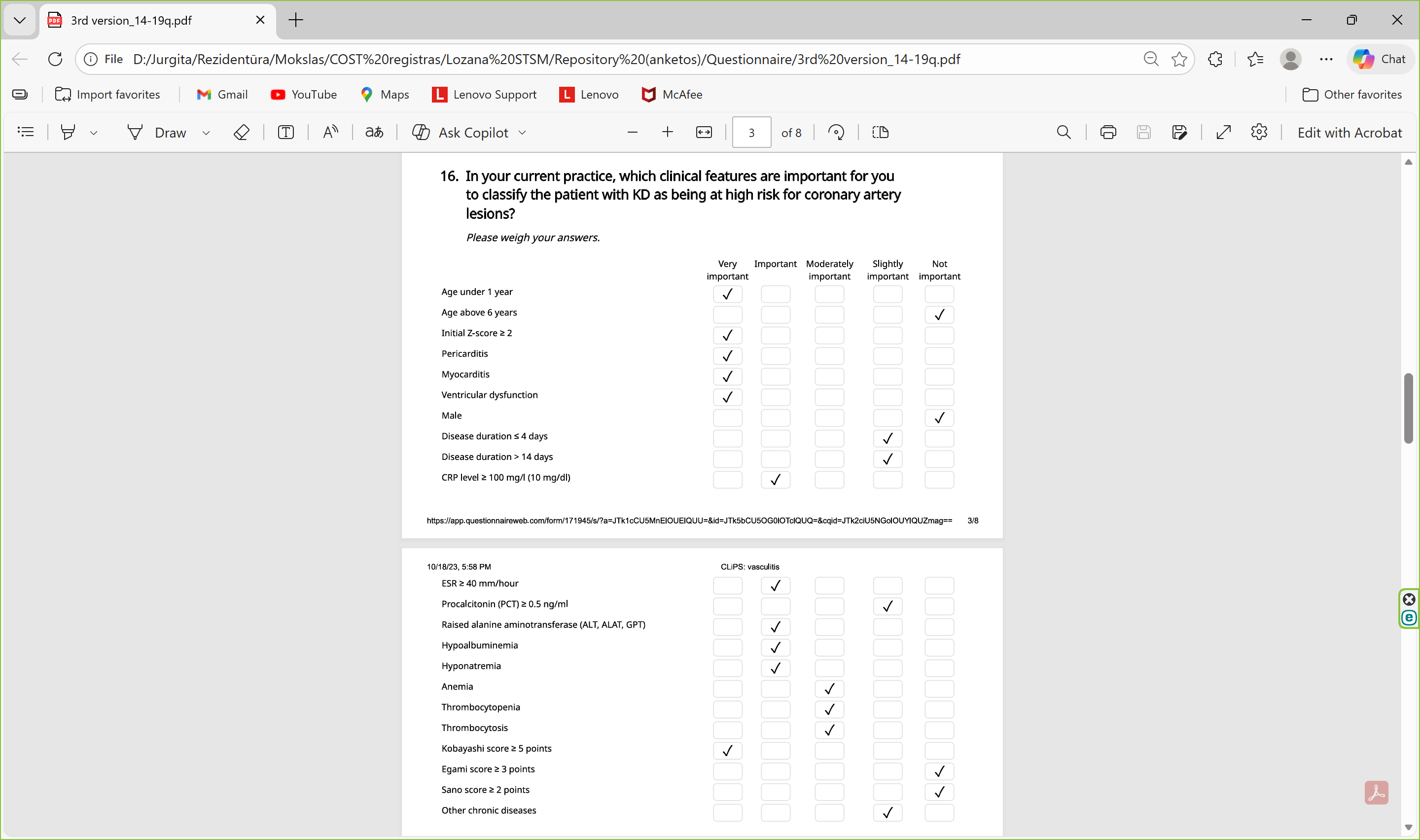

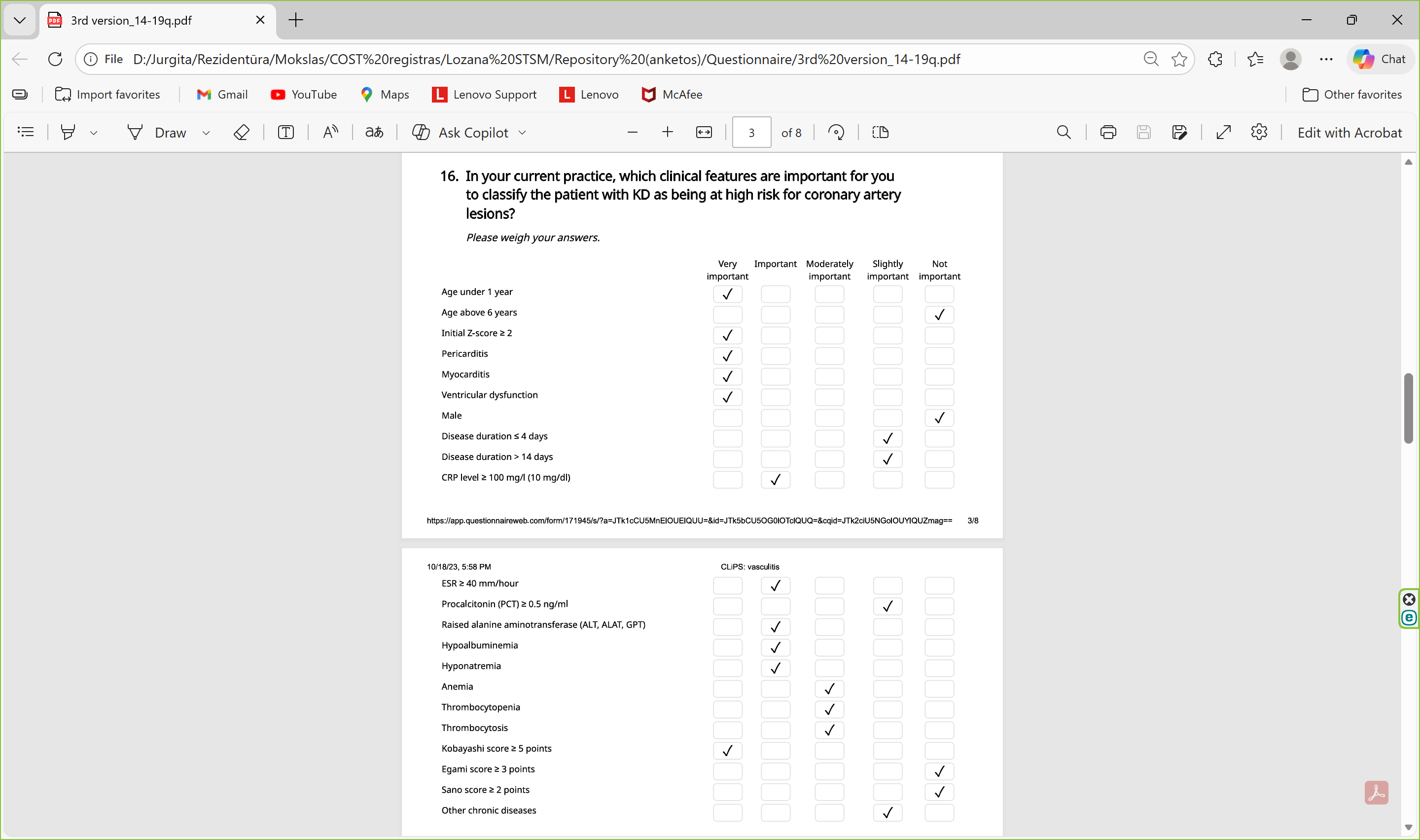

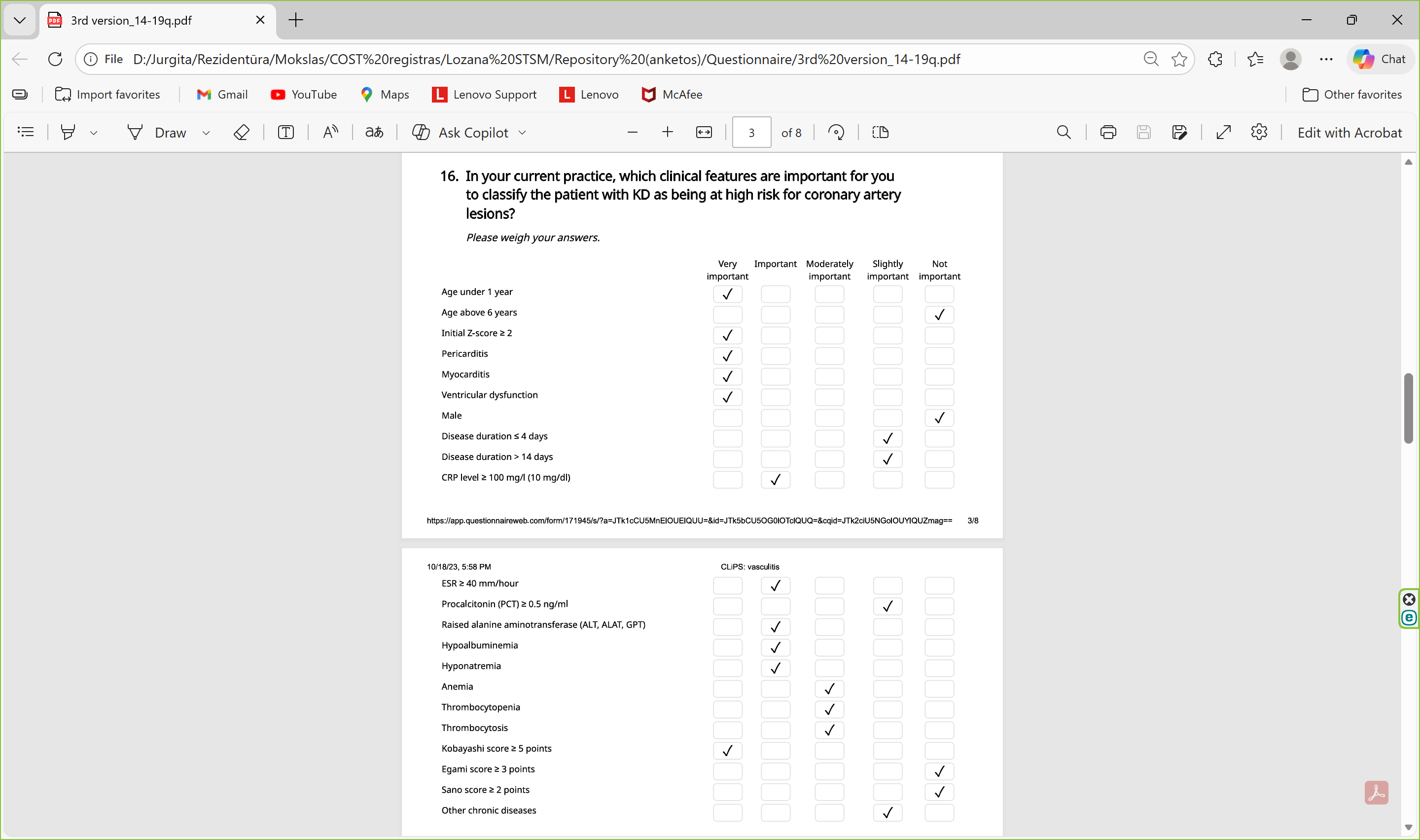

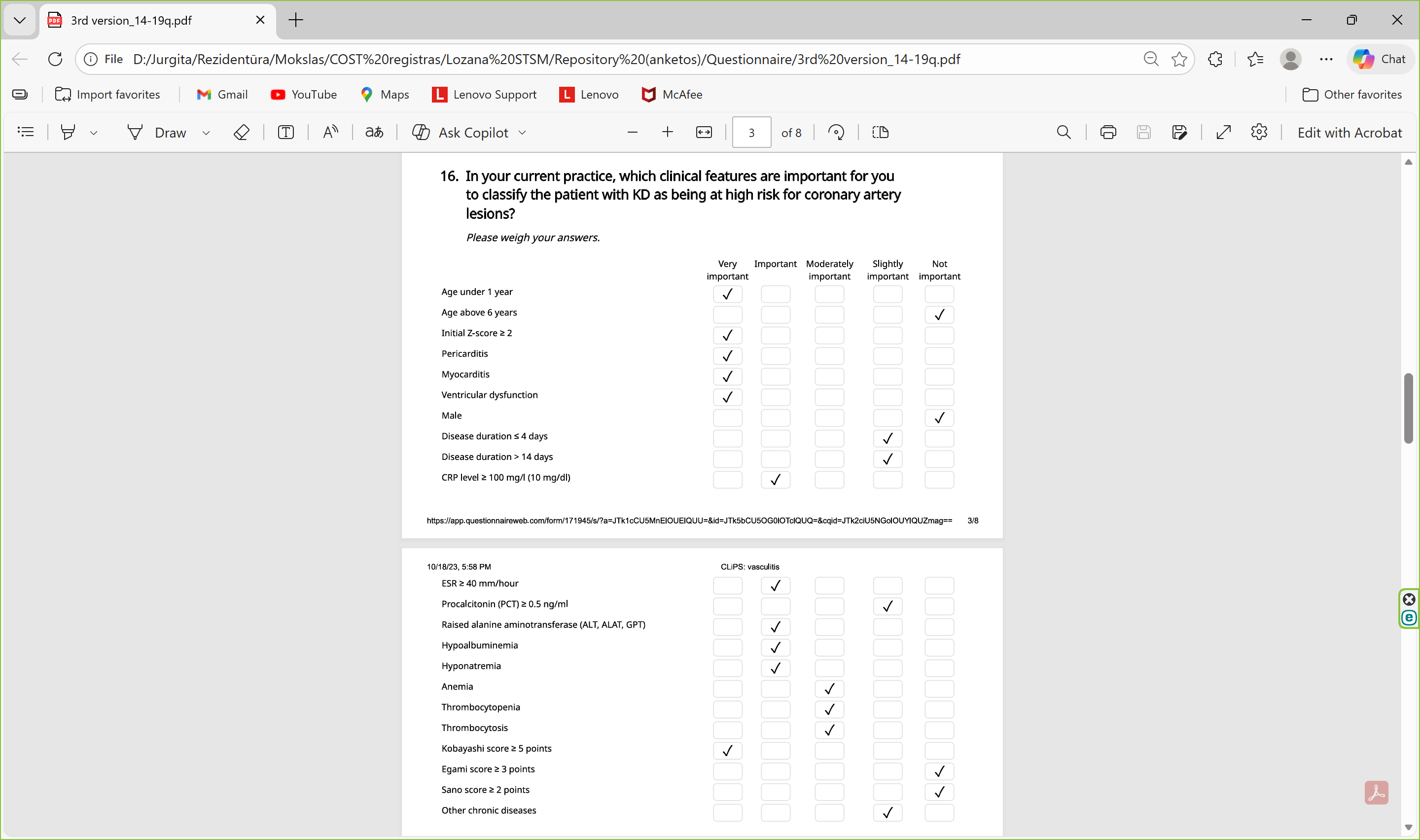

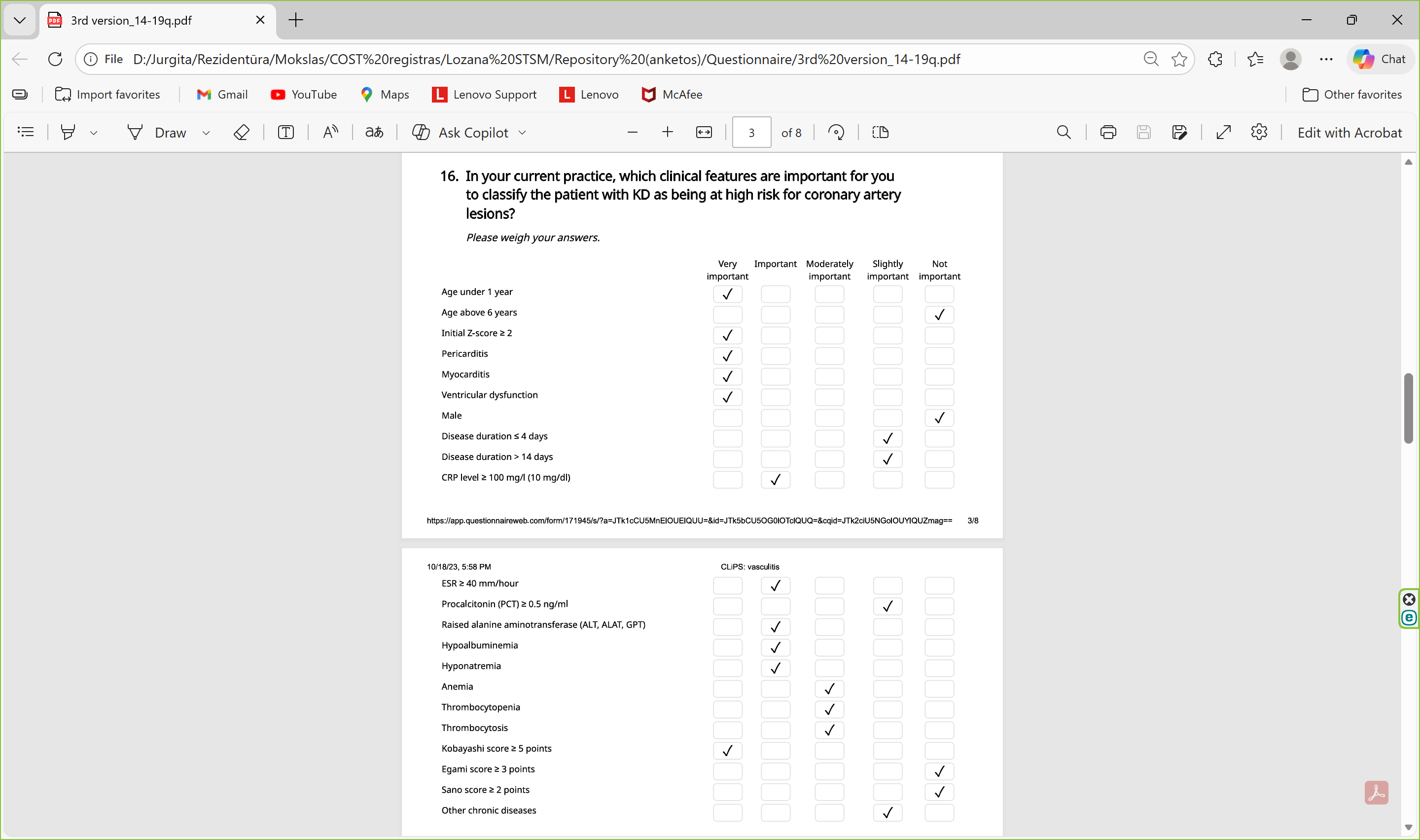

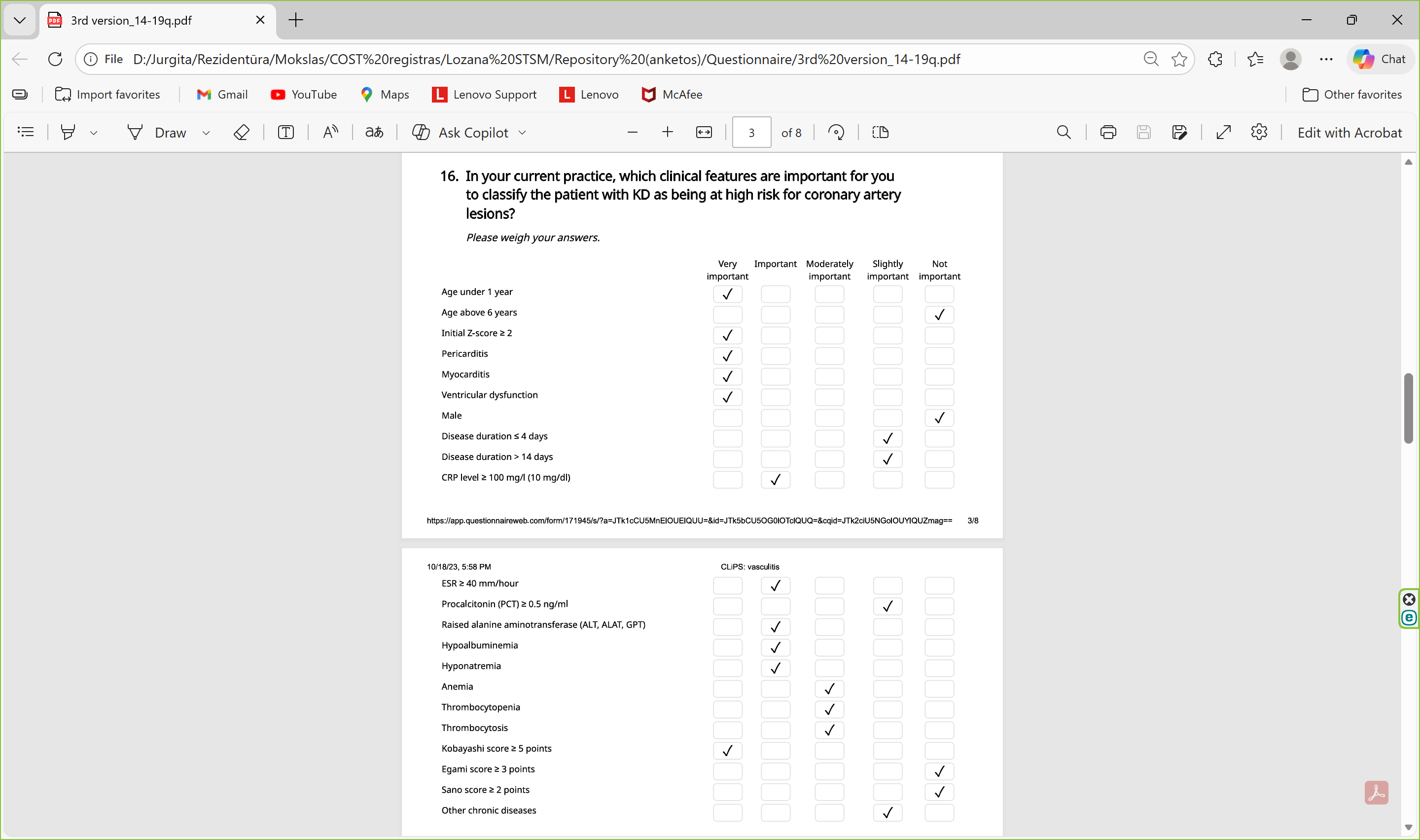

Supplement: keag340_Supplementary_Data [file keag340_supplementary_data.zip › Supplementary data S1_questionnaire.docx]
